# Wearable Sensing for Personal Thermal Comfort in the Built Environment: A Systematic Review of the Gap from Sensing to Actuation

**DOI:** 10.3390/s26175640

**Published:** 2026-09-04

**Authors:** Yorgos Spanodimitriou, Khawaja Talha Ejaz, Giovanni Ciampi, Michelangelo Scorpio, Massimiliano Masullo, Antonio Rosato, Luigi Maffei, Sergio Sibilio

**Affiliations:** Department of Architecture and Industrial Design, University of Campania Luigi Vanvitelli, Abazia di San Lorenzo, 81031 Aversa, Italy; khawajatalha.ejaz@unicampania.it (K.T.E.); giovanni.ciampi@unicampania.it (G.C.); michelangelo.scorpio@unicampania.it (M.S.); massimiliano.masullo@unicampania.it (M.M.); antonio.rosato@unicampania.it (A.R.); luigi.maffei@unicampania.it (L.M.); sergio.sibilio@unicampania.it (S.S.)

**Keywords:** thermal comfort, wearable sensors, machine learning, physiological signals, human-centric monitoring

## Abstract

**Highlights:**

**What are the main findings?**

- Skin temperature and heart rate dominate, used in 66% and 59% of studies.
- Multimodal sensing shows higher accuracy, but not uniformly across tasks.

**What are the implications of the main findings?**

- Validation strategy affects accuracy more than algorithm choice.
- Only 5 of 117 studies reach wearable-informed building control stage.

**Abstract:**

Wearable sensors enable continuous, non-invasive monitoring of physiological and near-body environmental parameters, offering an occupant-centric alternative for thermal comfort assessment and building control. This systematic review analyses 117 studies (2008–2026) using body-worn sensors, tracing them along a four-stage pipeline: sensing, signal integration, comfort modeling, and building actuation. This reveals a previously unquantified bottleneck: while all 117 studies perform sensing and 83 (71%) integrate physiological and environmental signals, only 52 (44%) build predictive models and just five (4%) reach the building-actuation stage, of which only three implement closed-loop, wearable-informed control. Skin temperature (77 studies, 66%) and heart rate (69, 59%) are the most monitored signals, predominantly at the wrist (70, 60%); multimodal configurations are associated with higher accuracy than single-domain approaches. Machine learning models reach median classification accuracies near 90%, though validation strategy matters: leave-one-subject-out reaches 85% versus 90% for within-subject k-fold. Five structural limitations are identified: small, homogeneous samples (median 16 participants), laboratory-dominated designs (73 studies, 62%), inconsistent validation, limited open data, and unresolved multi-occupant aggregation. The field has learned to measure the occupant but not yet to act on the measurement. Closing this gap requires open benchmark datasets, standardized validation including leave-one-subject-out testing and PMV benchmarking, transfer learning, multi-occupant integration, and inclusive recruitment of vulnerable populations.

## 1. Introduction

Buildings account for approximately 30% of global final energy consumption, with heating, ventilation, and air-conditioning (HVAC) systems responsible for roughly 38% of this demand [1,2,3]. A substantial share of this energy is spent maintaining indoor conditions intended to satisfy thermal comfort requirements defined by standards such as ASHRAE 55 [4] and ISO 7730 [5]. Nevertheless, evidence from the ASHRAE Global Thermal Comfort Database II, comprising over 100,000 field observations, indicates that occupant dissatisfaction rates persistently hover around 40% even in code-compliant buildings [6,7]. This disconnect between energy expenditure and actual occupant satisfaction points to a structural limitation in how thermal comfort is modeled and delivered.

Traditional thermal comfort frameworks, whether the steady-state PMV/PPD model [8] or the adaptive model [9], rely on time-averaged environmental measurements and population-level assumptions about metabolic rate and clothing insulation. These approaches treat occupants as passive, interchangeable recipients of a uniform thermal stimulus. In practice, thermal perception is shaped by individual physiology, behavioral adaptation, psychological state, and the dynamic interplay between the body and its immediate microenvironment [10,11]. The consequences of this mismatch are not merely academic: overconditioned spaces waste energy while underconditioned spaces compromise health and cognitive performance [12,13]. A recent systematic review of personal thermal comfort models confirmed that aggregate models consistently fail to capture individual variability, motivating a paradigm shift toward data-driven, person-specific predictions [14].

Wearable sensing technologies offer a fundamentally different approach. By enabling continuous, non-invasive monitoring of physiological indicators (skin temperature—SKT, heart rate—HR, heart rate variability—HRV, electrodermal activity—EDA, and, in some cases, electroencephalography—EEG), wearables can capture thermoregulatory responses as they unfold [15,16]. When combined with compact near-body environmental sensors measuring air temperature, humidity, radiant conditions, and air velocity in the occupant’s immediate vicinity, these devices provide a high-resolution, dynamic picture of the person-environment interaction that fixed monitoring stations and sporadic subjective surveys cannot achieve [17]. A six-month longitudinal field study demonstrated that personal comfort models trained on wearable data (skin temperature, near-body temperature, and heart rate) achieved median F1-scores of 0.78 for thermal preference prediction, substantially outperforming aggregate models under the same conditions [7]. The sensing repertoire is widening beyond the established wearable modalities: optical and photonic approaches are extending the range of physiological information that can be acquired, reconstructing cardiac signals in the presence of motion artifacts and decoding subject-invariant affective information from them, and in doing so they bring into focus a methodological problem that recurs throughout this review, namely cross-individual generalization [18]. More recent studies integrating multimodal physiological and environmental features have reported classification accuracies exceeding 85–90% in controlled settings [19]. Those results, however, are obtained almost entirely under controlled conditions, and the distance between them and an operating building is the subject of this review.

Indeed, the trajectory from wearable data to actionable building control has been described as a three-stage pipeline [20]: sensing (acquisition of physiological and/or environmental data), processing (signal cleaning, feature extraction, and data fusion), and modeling (classification or regression of comfort states). The output of this pipeline can, in principle, inform real-time HVAC control strategies that respond to individual occupant needs rather than zone-averaged setpoints. Recent implementations of occupant-centric control using personal comfort models have demonstrated energy savings of 10–30% while simultaneously improving satisfaction [21,22]. This vision of data-driven, responsive environmental control represents a paradigm shift from prescriptive to adaptive building operation.

Despite the rapid growth of research in this area, several critical gaps persist. Most studies rely on small, demographically homogeneous samples (typically young university students), limiting the generalizability of their findings [20,23]. Experimental conditions, comfort scales, sensing configurations, and modeling approaches vary widely across studies, making cross-study comparison difficult. Validation protocols range from simple train-test splits to rigorous leave-one-subject-out cross-validation. Yet, this heterogeneity is rarely discussed in a way that allows practitioners to assess the transferability of reported performance metrics [20]. Furthermore, the pathway from comfort prediction to actual HVAC integration remains largely theoretical, with only a handful of studies demonstrating closed-loop implementations [24].

### 1.1. Previous Review Works and Novelty

Considering these gaps, recent review papers have advanced the understanding of specific aspects of this landscape. Čulić et al. [25] surveyed smart monitoring technologies for personal thermal comfort, covering connected environmental sensors, camera-based systems, and wearable devices up to 2021. Their review provided a valuable taxonomy of sensing modalities but did not analyze predictive model performance or energy implications in a structured, quantitative manner. Gao and Ghahramani [20] examined the development of physiology-based personal comfort models through a sensing-preprocessing-modeling framework, identifying issues such as limited data cleaning and the underuse of unsupervised learning. However, their analysis was restricted to 28 indoor studies. It did not cover near-body environmental sensing, hardware characteristics, or the growing body of work on outdoor and urban thermal comfort. In this domain, wearable sensing is increasingly used to assess pedestrian exposure and to validate microclimate models [26]. Costantino et al. [27] systematically reviewed the use of off-the-shelf wearable sensing devices in thermal comfort research, providing a detailed framework of device types and physiological signals, but did not connect hardware characteristics to modeling performance or building energy outcomes. Mansi et al. [16] provided an overview of physiological signal processing for comfort research, focusing on biomedical signal extraction and sensor reliability, but did not extend the analysis to multi-domain integration or energy implications.

Collectively, these reviews have advanced the understanding of individual components of wearable-based comfort research: sensing hardware, physiological signal processing, and model development. Yet each treats these components in isolation, as parallel topics to be surveyed rather than as sequential stages of a single prediction-to-practice pipeline. When tracing the corpus longitudinally from raw signal acquisition through to building actuation, a gap emerges clearly: functioning tools accumulate at the sensing and modeling stages but stop at the point of closed-loop building integration. No prior review has quantified this gap or diagnosed where, and why, the pipeline breaks down.

Table 1 compares the present review with the most closely related prior contributions. This review addresses that gap by analyzing 117 studies as a single four-stage pipeline rather than as a set of parallel topics. The aim is to locate where wearable thermal comfort research stops going from prediction to practice by quantifying corpus coverage at each stage (sensing, signal processing, comfort modeling, and building actuation) and identifying the methodological and structural reasons. To the authors’ knowledge, this is the first review to treat the wearable thermal comfort literature as a single sensing-to-actuation pipeline rather than as a set of parallel research topics. Corpus coverage is quantified at each stage of the pipeline, the effect of validation strategy on reported accuracy is analyzed systematically across the classification studies of the corpus, the hardware landscape is linked to the sensing configurations, feature sets and modelling performance, and, finally, a structured diagnosis of the prediction-to-practice gap is carried out, from which a research agenda addressing benchmark data, validation standards, transfer learning, multi-occupant integration and inclusive recruitment is derived.

### 1.2. Structure of This Review

The review follows a PRISMA 2020-compliant protocol [28], with a reproducible Scopus search strategy, screening via ASReview LAB v3 [29], and structured data extraction across 24 standardized variables. The corpus, substantially larger than those of prior reviews in this domain, spans both indoor and outdoor/urban environments, includes studies with machine learning models and with statistical analyses, and covers publications without temporal restriction to capture both foundational and recent contributions.

The review is organized as follows: Section 2 describes the systematic methodology. Section 3 provides a quantitative overview of the corpus. Section 4 examines wearable sensing technologies, organized by signal domain. Section 5 analyzes the personal comfort modeling pipeline, from feature engineering to predictive performance, and presents a cross-study performance comparison and a validation strategy analysis. Section 6 discusses the integration of wearable-based comfort models with building systems and energy management. Section 7 identifies methodological limitations, research gaps, and future directions. Section 8 presents conclusions.

## 2. Materials and Methods

**Figure 1:**
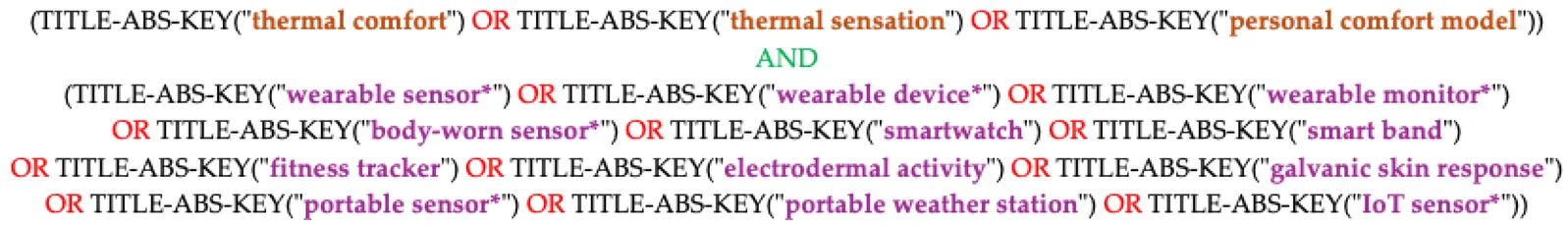
Primary Boolean query string used on the Scopus database. Terms highlighted in orange identify the main topics of the review, while terms highlighted in purple identify the technologies and sensing approaches in relation to these topics. The asterisk (*) denotes the truncation operator, allowing different word endings (e.g., singular and plural forms) to be retrieved without explicitly listing each variant.

This review was conducted following the Preferred Reporting Items for Systematic Reviews and Meta-Analyses (PRISMA) 2020 guidelines [28]. The methodology comprised four stages: literature identification, screening, eligibility assessment, and structured data extraction. The literature search was conducted on Scopus [30], selected for its comprehensive coverage of engineering, environmental science, and building technology research [31]. The single-database strategy was adopted deliberately, favoring the reproducibility of the Boolean queries, the record counts, and the screening decisions reported below over combining databases with heterogeneous indexing and export formats. The prior-knowledge seed set described below, assembled from the authors’ domain expertise independently of the database search, was fully contained within the retrieved corpus, indicating that the core literature of the field was captured. No temporal restriction was applied to the search in order to capture both foundational studies and recent developments. The review covers studies published up to March 2026, ensuring an up-to-date representation of the current state of the field. Two complementary queries were designed to maximize recall while maintaining precision. The primary query combined thermal comfort terminology with wearable and portable sensing terms (Figure 1).

**Figure 2:**
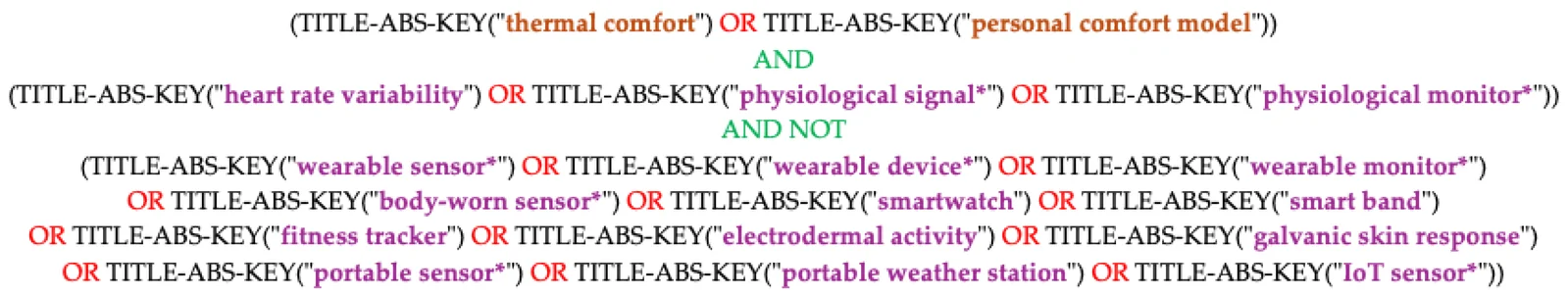
Supplementary Boolean query string used on the Scopus database. Terms highlighted in orange identify the main topics of the review, while terms highlighted in purple identify the technologies and sensing approaches in relation to these topics. The asterisk (*) denotes the truncation operator, allowing different word endings (e.g., singular and plural forms) to be retrieved without explicitly listing each variant.

This query returned 350 records. A supplementary query was designed to capture studies that used physiological sensing for thermal comfort without explicitly using the term “wearable” (Figure 2).

The “AND NOT” clause excluded records already retrieved by the primary query, returning an additional 151 unique records. After merging and deduplication, the combined corpus comprised 501 unique records. The screening based on titles and abstracts was performed using ASReview LAB [29], an open-source active learning platform validated for systematic reviews [32]. ASReview employs machine learning models that iteratively learn from the reviewer’s labeling decisions, presenting records in order of predicted relevance rather than randomly. This approach has been shown to reduce screening workload by up to 95% compared to manual screening while maintaining high recall [29,32]. A dual-round screening strategy was adopted to minimize the risk of missing relevant studies. In the first round, the ELAS Ultra model (TF-IDF feature extraction with Linear SVC classifier) was used.

The authors screened 190 records, identifying 69 as relevant and 121 as not relevant, and terminated screening after 34 consecutive irrelevant records. The rule applied is the n_consecutive_irrelevant stopping criterion implemented in ASReview, with its threshold set at 30, which is consistent with the heuristics evaluated in the methodological literature on stopping rules for active-learning screening, which converge on a fixed number of consecutive irrelevant records as a practical, if conservative, criterion [32,33,34,35]. However, it carries an inherent risk of false negatives. To overcome this risk, an initial seed set was defined, comprising 42 prior-knowledge papers, 38 labeled as relevant and 4 as not relevant, covering the core topics of the review (wearable physiological sensing, personal thermal comfort models, and wearable-informed HVAC control), selected by the authors based on their established familiarity with the domain.

Inter-rater reliability has been assessed on a random sample of 150 of the 501 retrieved records (30%), screened independently by two authors on title and abstract against the eligibility criteria. Observed agreement was 93.3%, and Cohen’s kappa was 0.84, which falls within the almost-perfect range of the conventional interpretation [36]. The ten disagreements were unidirectional: in each case one reviewer judged the record eligible, and the other did not, never the reverse, which indicates a difference in inclusiveness at the abstract stage rather than a divergent reading of the criteria. All disagreements were resolved by discussion between the two reviewers until consensus was reached.

The stopping criterion was then subjected to a sensitivity analysis in the strict sense, by continuing screening beyond the point at which the criterion had been met and recording whether further relevant studies emerged. At the point at which the criterion was met, 232 records had been screened (42 prior-knowledge records and 190 queried by the model), and 107 relevant records had been identified. A second round was conducted over all records not labeled as relevant in the first round—that is, both those marked as not relevant and those never presented—using the ELAS Heavy model (MXBAI dense embeddings with a support vector classifier under class-balanced re-weighting and a maximum-uncertainty query strategy). This transformer-based model, which captures semantic relationships that bag-of-words approaches may miss, helped identify 46 additional relevant records from unscreened records, recovering studies on topics such as thermal alliesthesia, psychophysiological responses, and pediatric populations that the first-round model had ranked lower. Applied on its own, the first-round stopping criterion would therefore have terminated screening with 107 of the 153 relevant records identified (70%), missing the 46 recovered in the second round. The consecutive-irrelevant rule was thus not, in this corpus, a sufficient stopping criterion. The result is attributable to the high prevalence of relevant records (153 of 501, 31%), since a fixed threshold of this kind is calibrated for corpora in which relevant records are rare. The screening performance of the two rounds combined can be quantified against the 115 relevant records that were not part of the prior-knowledge seed set, which are the records the active-learning procedure had to discover rather than being given. Half of them were identified within the first 161 records screened, 90% within 343, and 95% within 358 of the 501 records, corresponding to a work-saved-over-sampling [37] value at 95% recall of 24%. All 115 had been identified after 378 records, and the final 25 records screened yielded no further relevant study. 92 records were never presented to a reviewer in either round; ranked by the final second-round model, these occupy positions outside the range in which relevant records were still being identified. The complete screening history, including the labeling order, the model configuration of each round and the final ranking, is available in the ASReview project file provided as Supplementary Material.

Across both rounds, the screening yielded 153 relevant records (including prior knowledge seeds) and 348 irrelevant records. Post-screening quality checks resulted in three records being reclassified from irrelevant to relevant and two review papers being excluded, bringing the total to 154 records forwarded to full-text assessment.

At this stage, studies were included if they satisfied both of the following conditions: (i) the study employed wearable or body-worn devices to collect physiological data (e.g., skin temperature, heart rate, and electrodermal activity) and/or near-body environmental data (e.g., air temperature, humidity, and air velocity at the occupant level); and (ii) the study investigated the relationship between these data and human thermal comfort, thermal sensation, or thermal preference. Studies were excluded if they fell into any of the following categories: purely clinical or medical studies where wearable sensors served diagnostic purposes unrelated to thermal comfort; studies relying exclusively on fixed environmental sensors without any wearable or body-worn component; studies using only camera-based systems (e.g., infrared thermography) without wearable sensing; studies conducted exclusively in vehicles (trains, cars, aircraft); studies focused on personal comfort system (PCS) actuators rather than sensing; review papers, meta-analyses, editorials, or conference abstracts without full-text availability; simulation-only studies without human participants. When both a conference paper and a journal paper reported the same dataset, the journal version was retained.

The final corpus was consolidated to 117 entries, with the full text retrieved for these studies. Data extraction was performed using a structured protocol across 24 variables organized into five categories:

- Study identification (authors, year, title, journal, keywords);
- Study design (country, study type, environment, primary domain, sample size);
- Sensing configuration (wearable devices, body locations, physiological signals, near-body and fixed environmental sensors, sampling rate);
- Subjective assessment (comfort scales, scale type, survey frequency);
- Modeling approach (algorithm, input features, output variables, validation methodology, validation results).

Study quality was assessed alongside the extraction as a set of ten binary indicators (yes/no) applied uniformly to all 117 studies, each capturing one methodological or reporting condition whose absence has an identifiable consequence for the interpretation of the reported results:

- Sample size of at least 30 participants;
- A setting other than the laboratory;
- Use of near-body environmental sensing;
- A reported sampling rate;
- Cross-subject validation, where a predictive model is developed;
- Comparison against a conventional comfort index;
- Reporting of participant age and sex;
- Open availability of data or code;
- A quantitative figure for the proportion of data retained or discarded after preprocessing;
- Reported ethical approval.

**Figure 3:**
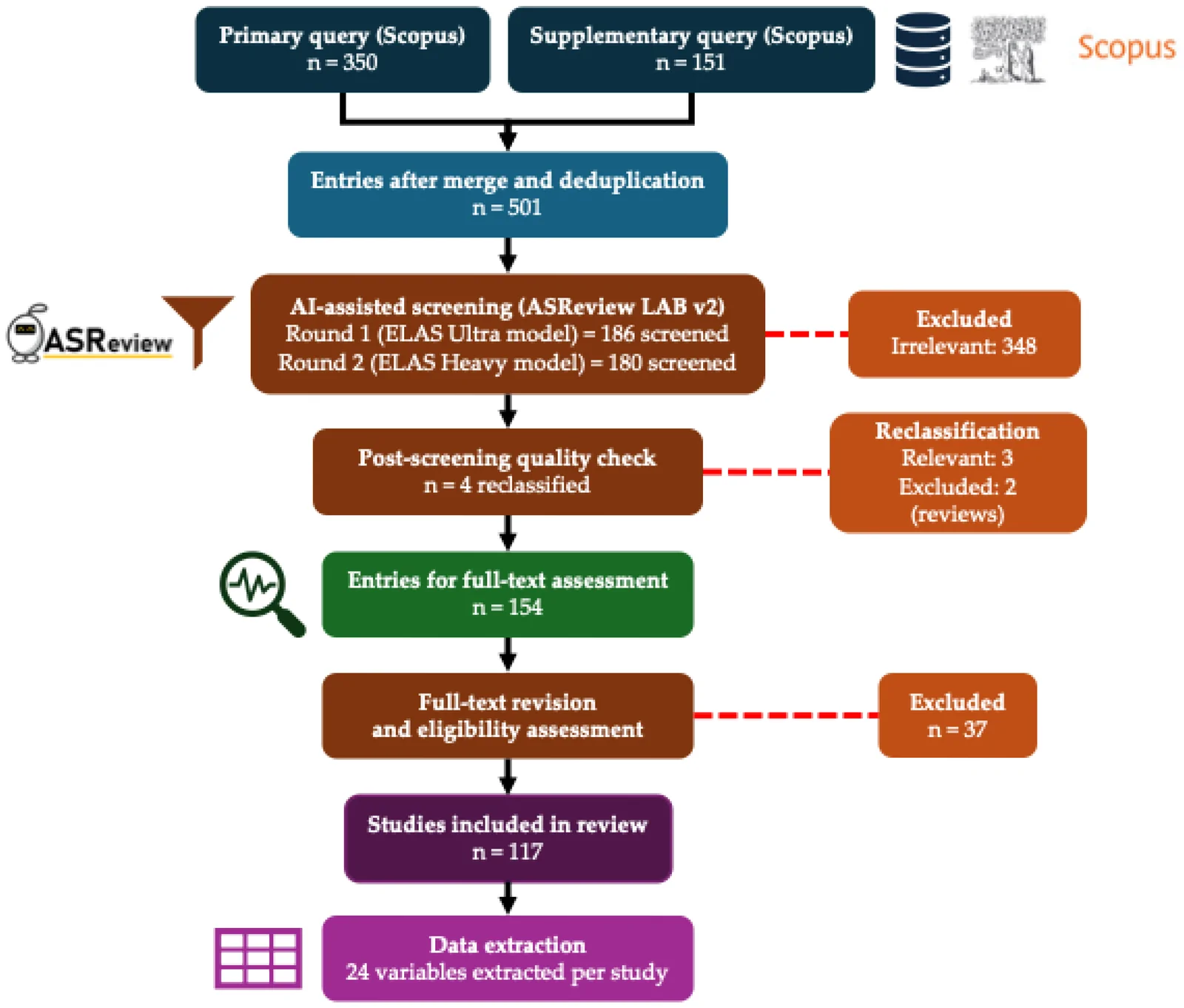
PRISMA flow diagram as implemented in this study.

Figure 3 presents the PRISMA flow diagram summarizing the whole process, while the complete extraction dataset (24 variables + 10 quality binary indicators) is provided as Supplementary Material and, with reduced detail, in Appendix A, to support reproducibility and future meta-analyses.

## 3. Overview of the Studies

The final corpus comprises 117 studies published between 2008 and 2026, spanning 37 distinct publication venues. This section provides a quantitative profile of the reviewed literature, organized by temporal trends, geographic distribution, study design, participant characteristics, sensing configurations, and modeling approaches.

**Figure 4:**
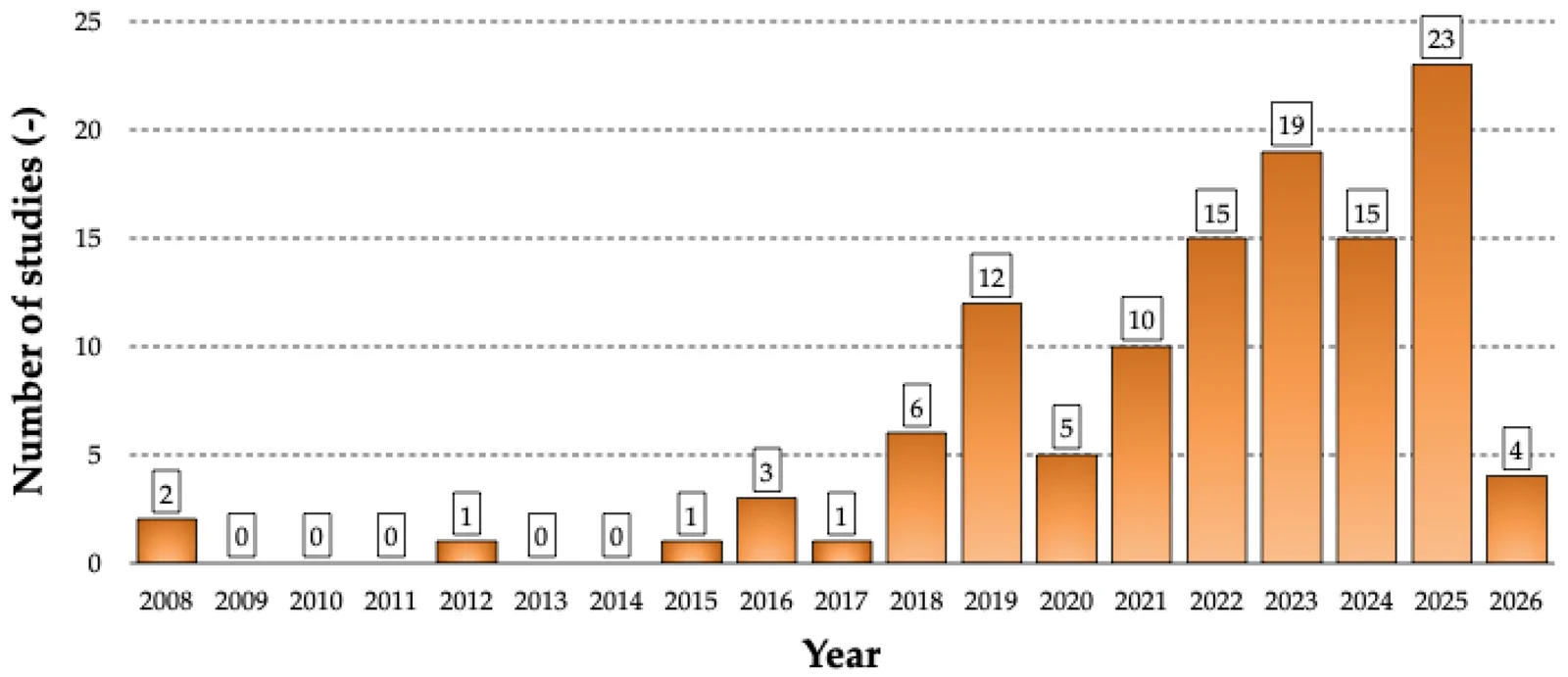
Number of studies per year.

In terms of numbers, publication output has grown markedly over the review period, with a pronounced acceleration from 2019 onward (Figure 4). The corpus includes isolated foundational studies from 2008 and 2012, followed by a gradual increase since 2015. Output rose sharply from 2019 (12 studies), sustained high levels through 2022 (15 studies) and 2023 (19 studies), and peaked in 2025 (23 studies), with four publications from 2026 captured at the time of the search. This growth trajectory mirrors two concurrent developments: the commercial maturation of affordable, research-grade wearable platforms (most notably the Empatica devices [38], which received FDA clearance for adults in 2018 and for children in 2019) and consumer smartwatches with PPG and EDA capabilities, and the parallel expansion of machine learning frameworks suitable for physiological time-series classification [27].

**Figure 5:**
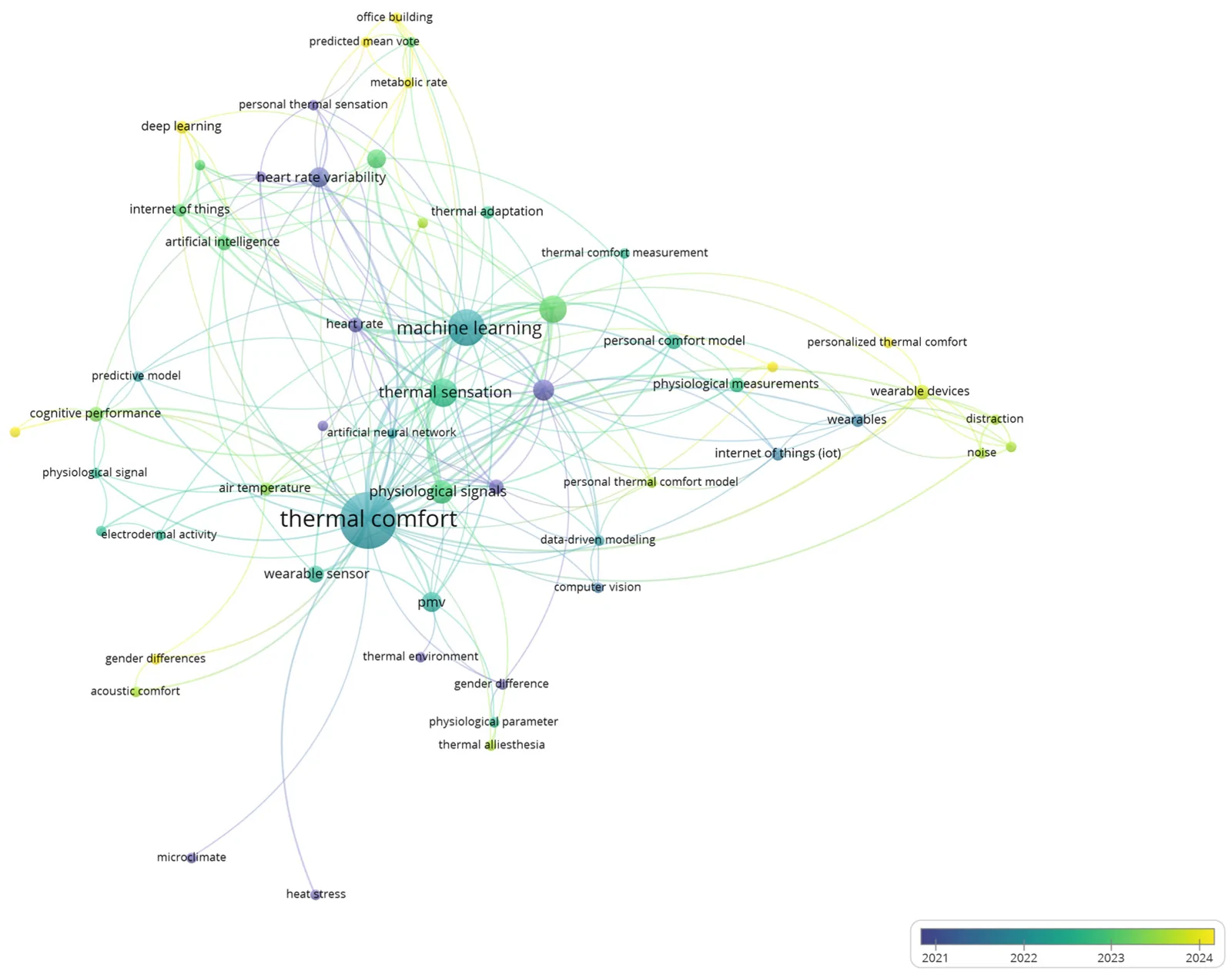
Visualization of the keywords’ network. The size of each circle is proportional to the frequency of occurrence of the corresponding keyword, with larger circles indicating more frequently occurring keywords.

A keyword co-occurrence analysis of the corpus using VOSviewer v1.6 [39] reveals the field’s thematic landscape (Figure 5).

In Figure 5, the overlay visualization, colored by average publication year, reveals a clear temporal gradient across the keyword network. The earliest terms (around 2020) are foundational sensing and comfort concepts: “skin temperature” (eight occurrences), “heart rate”, and “wearable device” anchor the network’s core alongside the dominant hub “thermal comfort” (54 occurrences, total link strength 99). The mid-period layer (2022) includes the methodological backbone of the field: “machine learning” (23 occurrences, link strength 66) emerges as the second-largest node, closely linked to “thermal sensation” (14 occurrences), “physiological signals” (9 occurrences), and “PMV” (7 occurrences). The most recent terms (>2023) occupy the network’s periphery and reflect emerging research directions: “deep learning”, “EEG”, “metabolic rate”, and “personalized thermal comfort” signal the field’s push toward more sophisticated signal processing and individualized models, while “noise” and “distraction” together with “occupant behavior” indicate the expansion of wearable comfort research beyond thermal sensing into broader indoor environmental quality. The cluster analysis identifies ten keyword communities, which can be interpreted as five thematic groupings: (i) core physiological sensing (skin temperature, heart rate, thermal sensation, EEG, and physiological measurements), (ii) machine learning and data-driven modeling (machine learning, physiological signals, and wearable sensors), (iii) AI, IoT, and smart building integration (artificial intelligence, deep learning, internet of things, occupant behavior, and noise/distraction), (iv) conventional comfort modeling and thermal physiology (PMV, thermal comfort, wearable sensor, thermal alliesthesia, and heat stress), and (v) HRV-based and office-focused applications (heart rate variability, cognitive performance, metabolic rate, and office building). This structure confirms the multidisciplinary nature of the domain, bridging physiology, computer science, and building engineering, and motivates the cross-domain organization adopted in the subsequent sections of this review.

**Figure 6:**
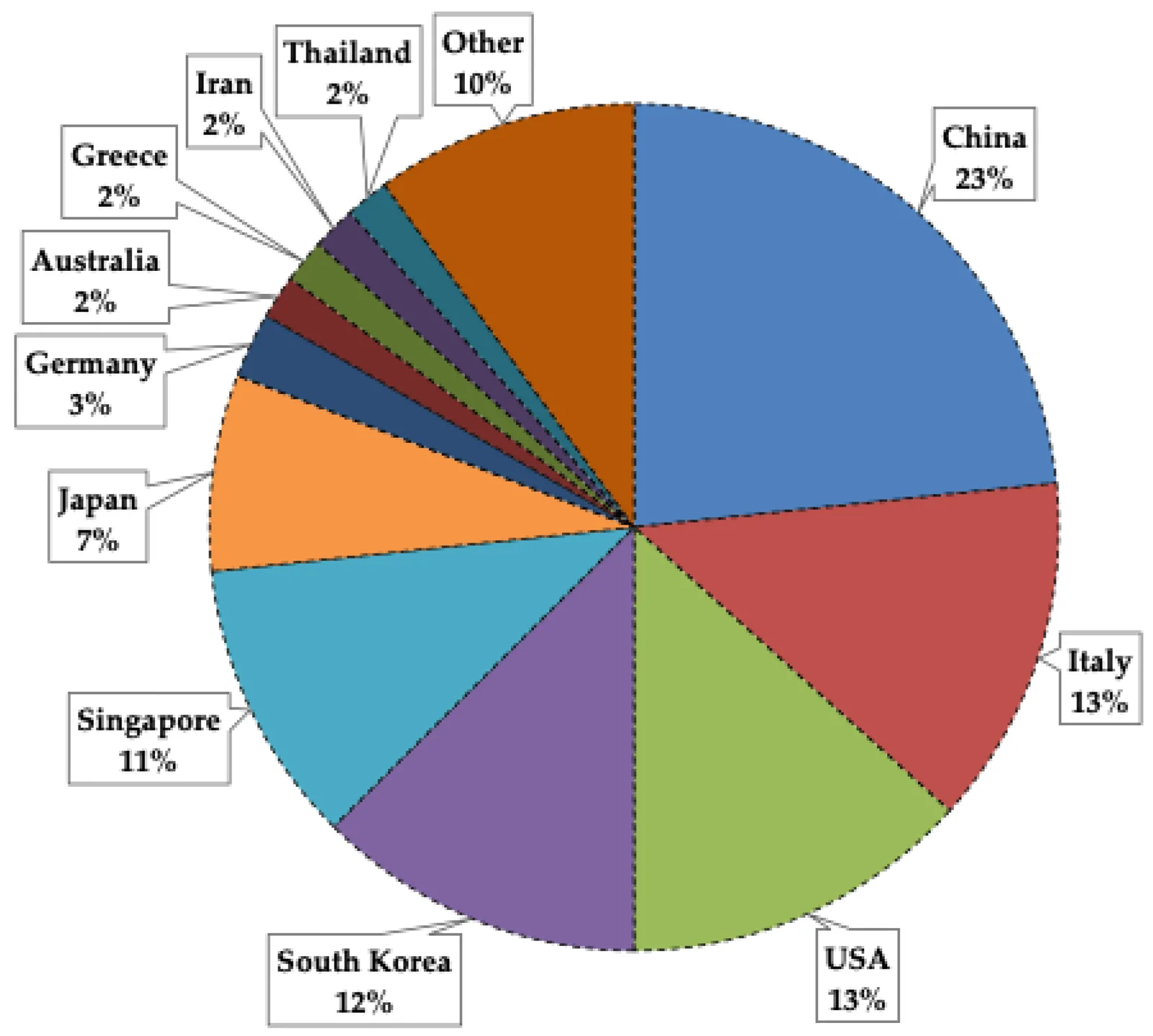
The corpus of this review organized by authors’ countries of origin.

In terms of geographical clustering, the corpus spans 23 countries across five continents, though with substantial geographic concentration (Figure 6).

China leads with 28 studies (23%), reflecting sustained investment in smart building research and large-scale field campaigns across diverse climate zones. Italy and the United States follow with 16 studies (13%) each, South Korea with 15 (12%), Singapore with 13 (11%), and Japan with 9 (8%). Together, these six countries account for 83% of the corpus. The remaining 17% is distributed across 17 countries, with small clusters in Germany (3), Australia, Greece, Iran, and Thailand (2 each), and single contributions from Belgium, Brazil, Canada, Croatia, Denmark, Egypt, Ghana, Malaysia, Mexico, the Philippines, Serbia, and Spain. The near-absence of research from Sub-Saharan Africa (one study, from Ghana) and South America (one study, from Brazil) represents a critical geographic blind spot. These regions face disproportionate heat-related health burdens, and the distinct thermoregulatory profiles associated with tropical, equatorial, and high-altitude climates remain essentially unrepresented in the wearable comfort literature.

**Figure 7:**
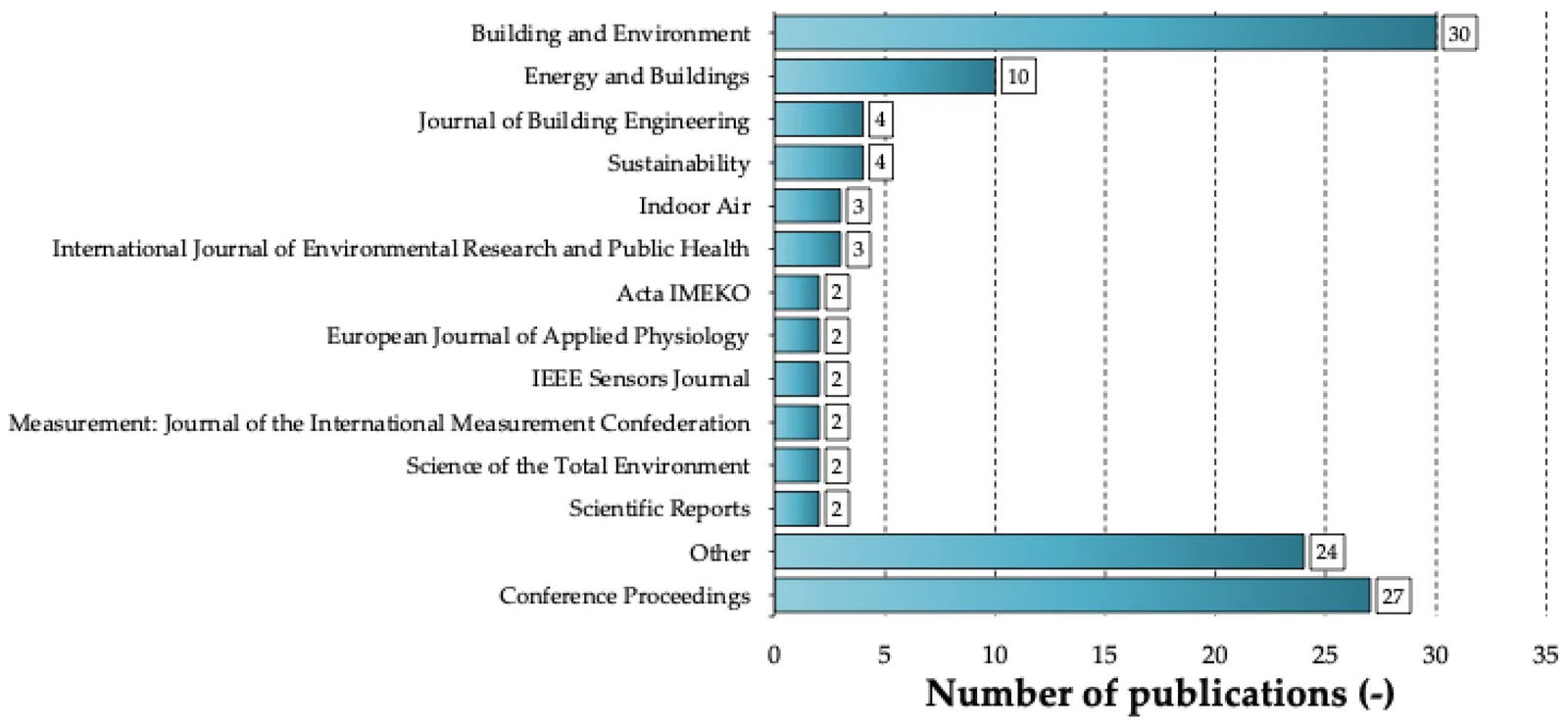
Distribution of the corpus by source type and journal.

The analysis of the dominant journal reveals the field’s building science orientation. As reported in Figure 7, *Building and Environment* hosts 30 studies (26%), *Energy and Buildings* hosts 10 (9%), and *Journal of Building Engineering* hosts four (3%), collectively accounting for 38% of the journal publications. Conference proceedings account for 27 entries (23%), a non-negligible share that indicates the field is still consolidating from emerging contributions into fully peer-reviewed literature.

**Figure 8:**
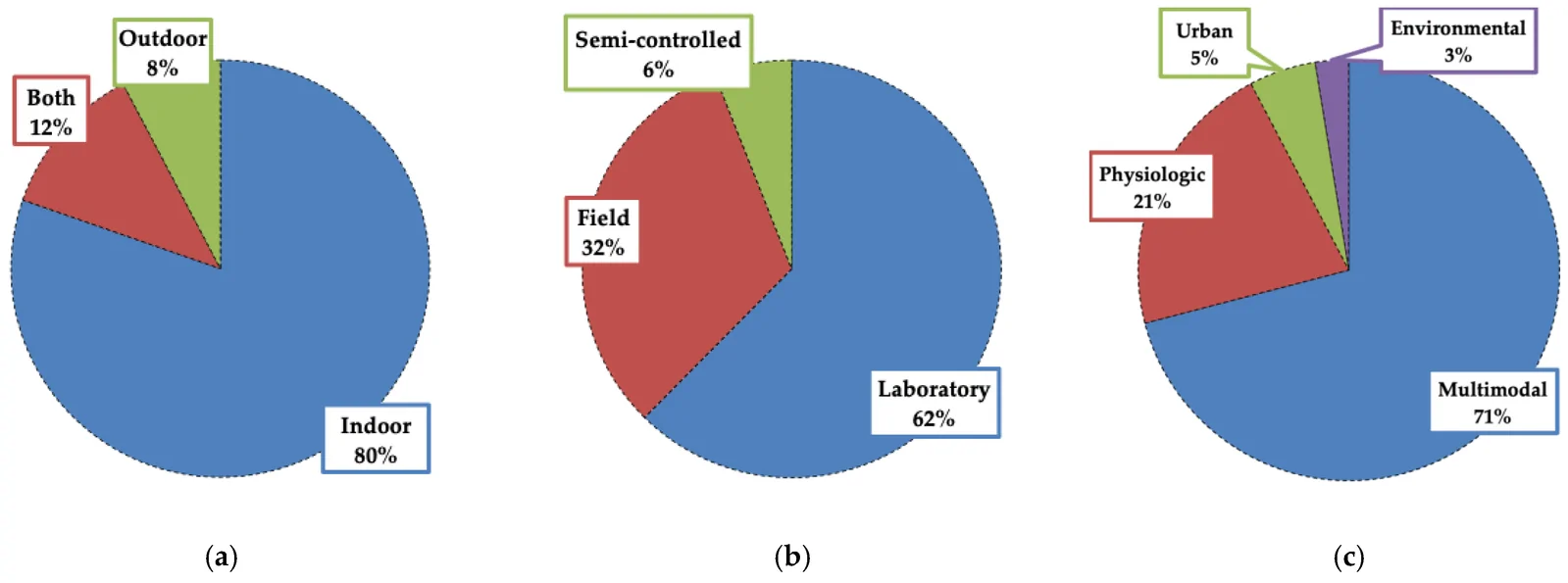
Corpus grouped in terms of: (**a**) domains, (**b**) study designs, and (**c**) scopes.

In terms of domains (Figure 8a), indoor studies dominate the corpus (94 studies, 80%), followed by studies covering both indoor and outdoor environments (14, 12%) and outdoor-only studies (9, 8%). Among study designs (Figure 8b), laboratory or climate chamber experiments constitute the largest group (73 studies, 62%), followed by field studies conducted in real-world and outdoor environments (37, 32%), and semi-controlled settings (seven, 6%). The predominance of laboratory studies reflects the methodological advantages of controlled thermal stimuli, standardized protocols, and reduced confounding variables, at the cost of ecological validity: laboratory conditions (with fixed clothing, constrained postures, limited behavioral adaptation, and short exposure durations) may not represent the complexity of real-world thermal experience [26]. From this point of view, the 37 field studies provide higher external validity but face challenges, including motion artifacts, variable compliance with wearable protocols, and uncontrolled environmental fluctuations. Moreover, as reported in Figure 8c, each study was classified into one of four primary domains based on its predominant contribution: (i) multimodal (83 studies, 71%), combining physiological and environmental sensing, (ii) physiological-only (25, 21%), focused on body-worn physiological monitoring, (iii) urban/outdoor (six, 5%), centered on pedestrian thermal exposure and microclimate assessment, and (iv) environmental-only (three, 3%), using body-worn environmental sensors without physiological measurement. The dominance of multimodal studies reflects the field’s recognition that thermal comfort is an inherently multidimensional construct requiring concurrent measurement of both human and environmental parameters [26].

Study durations and sample sizes vary substantially. Nearly half of the corpus (48 studies, 41%) involves single-session or multi-hour exposures, typically 30 to 120 min in climate chambers. A further 14 studies (12%) span 1–7 days, 21 (18%) extend to 1–4 weeks, and 9 (8%) exceed one month. The remaining studies do not report duration. Sample sizes across the corpus range from one to 640 participants. The literature is dominated by small and medium samples: 33 studies (28%) enrolled 10 or fewer participants, 40 (35%) enrolled between 11 and 20 participants, 29 (25%) enrolled between 21 and 50 participants, and only 15 (13%) enrolled 51 or more participants. The median sample size is approximately 16 participants. These patterns have direct consequences for the reliability of the findings. For the 52 studies employing machine learning models, where training data volume critically affects generalization, the prevalence of small datasets may raise concerns about overfitting, particularly when combined with high-dimensional feature spaces [23]. Only 23 studies (20%) provide any justification for their sample size, whether through formal power analysis, reference to comparable work, or post hoc assessment. This represents a significant methodological gap that limits the interpretability and comparability of reported model performance. Equally concerning is the demographic composition of the participant pools. Available data indicate a strong bias toward young adults (typically 20–30 years old) recruited from university populations, a demographic that is not representative of the broader building occupant population. Only seven studies (6%) explicitly include vulnerable or underrepresented populations, including four involving children and three involving elderly participants. Given that thermoregulatory capacity varies substantially with age (i.e., older adults exhibit reduced peripheral vasomotor control and sweat response, while children have higher metabolic rates per unit body mass [40,41]), the near-absence of targeted research on these groups has implications for both model generalizability and health-protective building design.

**Figure 9:**
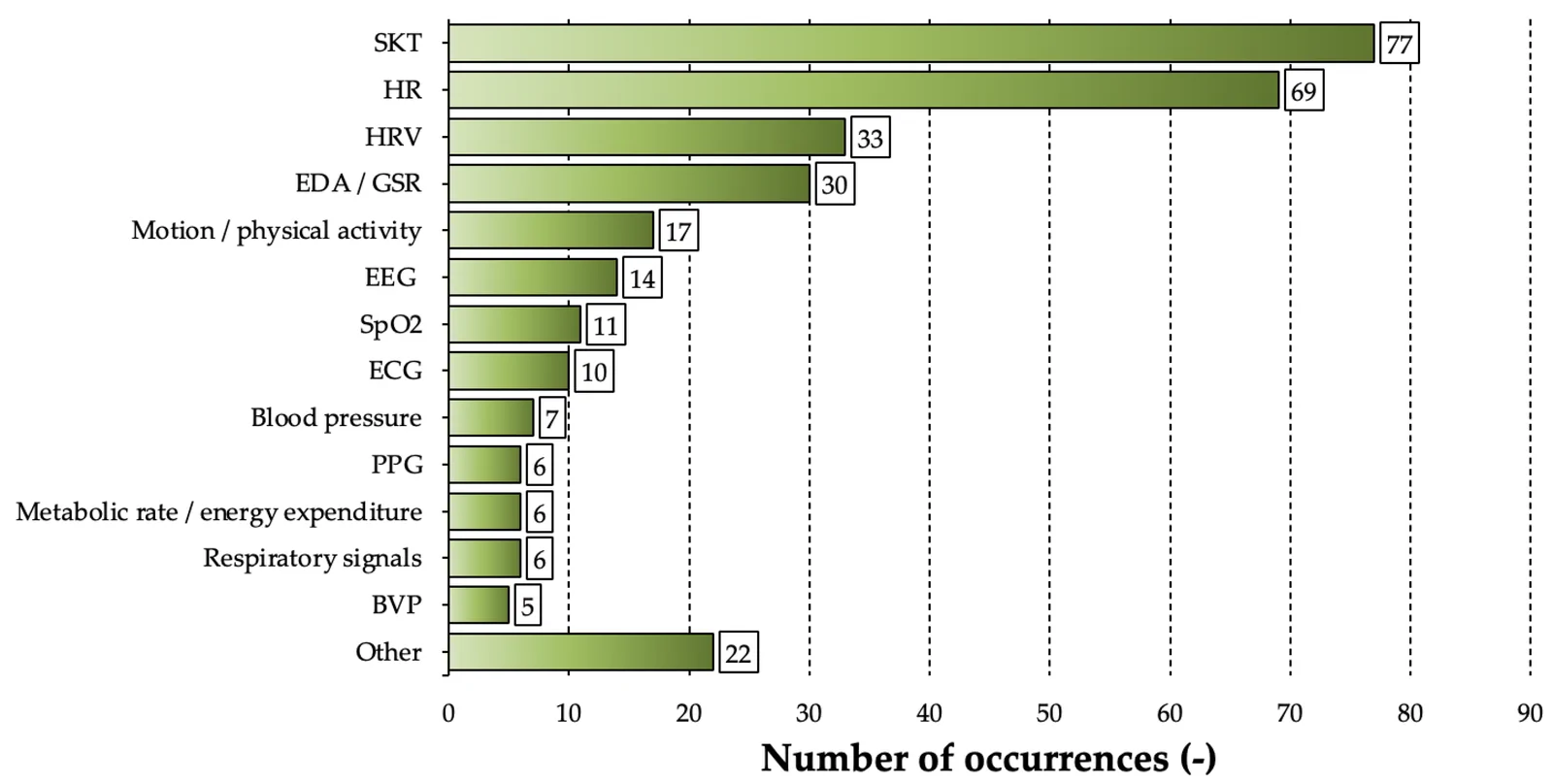
Number of occurrences for each physiological signal.

In terms of physiological signals (Figure 9), the most frequently monitored are skin temperature (SKT, 77 studies, 66%), heart rate (HR, 69 studies, 59%) and heart rate variability (HRV, 33 studies, 28%), electrodermal activity (EDA/GSR, 30 studies, 26%), and accelerometry/motion (17 studies, 15%). Less commonly measured signals include electroencephalography (EEG, 14 studies), blood oxygen saturation (SpO_2_, 11 studies), electrocardiography (ECG, 10 studies), blood pressure (seven studies), photoplethysmography (PPG, six studies), metabolic rate estimates (six studies), respiratory rate (six studies), and blood volume pulse (BVP, five studies). Isolated studies also capture perspiration rate, stress level, salivary cortisol, heat flux/loss, fEMG, and blood glucose (aggregated in “Other”, Figure 9).

The dominance of SKT and HR/HRV reflects both their established physiological relevance to thermoregulation, as direct markers of vasomotor and cardiovascular responses to thermal load [42,43,44], and the practical accessibility of commercial wrist-worn devices that capture these signals with minimal participant burden. The growing adoption of EDA (from near-zero before 2019 to 28 studies by 2025) is notable (and, as previously said, arguably interconnected with the widespread use of commercial wearable platforms, such as Empatica), as electrodermal activity provides a direct index of sympathetic nervous system activation linked to thermal stress and discomfort [45,46,47].

In terms of body locations for wearable sensing, the wrist is the most common measurement site (70 studies, 60%), followed by the head/forehead/ear (42, 36%), chest/torso (35, 30%), arm/forearm (27, 23%), lower limbs (22, 19%), and hand/finger (23, 20%). Less frequent locations include the back and shoulders (13 studies) and neck (five). Many studies employ multi-site configurations, as reflected in 301 body-location assignments across 117 studies (mean 2.6 sites per study). In particular, multi-site skin temperature measurements enable the estimation of mean skin temperature gradients, which are more predictive of thermal sensation than single-point measurements [44,48,49,50].

**Figure 10:**
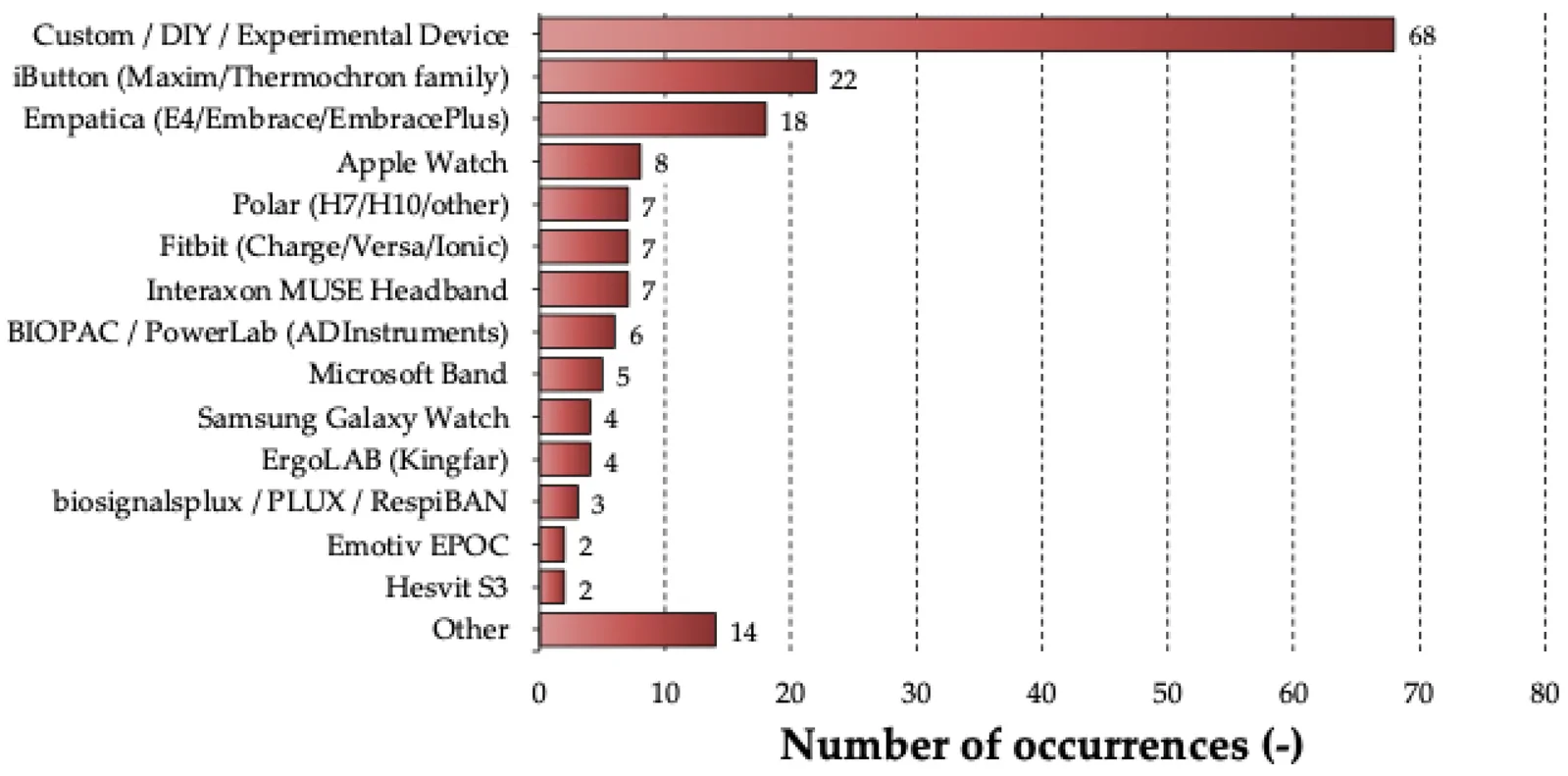
Number of occurrences for each wearable device family.

In terms of devices, the landscape is heterogeneous, as reported in Figure 10. A substantial number of studies employ custom-built, prototype, or non-mainstream devices, reflecting the prevalence of sensor platforms tailored to specific research needs and, indirectly, the limitations of existing commercial devices in meeting all research requirements simultaneously. Among commercial devices, the iButton logger family [51] (22 studies) and the Empatica E4/Embrace wristband [38] (18 studies) are most widely adopted, followed by Apple Watch [52] (eight studies), Polar chest straps [53] (seven studies), Fitbit devices [54] (seven studies), and the Muse EEG headband [55] (seven studies). Research-grade systems (e.g., BIOPAC/PowerLab [56,57]) (six studies), Microsoft Band [58] (five studies), Samsung Galaxy Watch [59] (four studies), and ErgoLAB [60] (four studies) account for additional shares.

In particular, the prominence of the Empatica E4 [38] has led to partial de facto standardization of multimodal sensing configurations, facilitating cross-study comparisons within this device subset. The iButton family [51], conversely, provides temperature logging, but its the standard for multi-point skin temperature measurement in field studies.

In terms of modeling approaches, 52 studies (44%) employ machine learning (ML) models for comfort prediction or classification, while 65 (56%) rely on statistical methods, including correlation analysis, regression, ANOVA, or descriptive statistics. Among ML studies, the most frequently adopted algorithm families at the study level are Support Vector Machines (SVMs, 31 studies), Random Forest and Extra Trees (29), Artificial Neural Networks (ANN/MLP/DNN, 19), k-nearest neighbors (KNN, 16), regression-based and linear discriminant methods (15), and decision trees (11). Gradient boosting methods (XGBoost, LightGBM, and CatBoost) appear in nine studies, while deep learning architectures (CNN—eight studies, LSTM/GRU/RNN—eight studies) are growing but remain less common. These frequencies reflect study-level counts; many studies benchmark multiple algorithms on the same dataset, with ensemble methods (Random Forest, gradient boosting) and SVM most frequently emerging as top performers. In terms of validation, the most common approach is k-fold cross-validation (28 studies), followed by hold-out train/test split (12 studies) and leave-one-subject-out (LOSOCV) cross-validation (8 studies). LOSOCV is particularly important in personal comfort modeling, as it tests whether a model trained on other individuals can predict a new person’s comfort, a fundamentally harder but more realistic evaluation [61,62,63,64,65].

Regarding the connection to building energy management, 80 studies (68%) discuss HVAC or energy-saving implications of their findings. However, the depth of this discussion varies considerably (from brief mentions in the conclusions to substantive analysis of control strategies). A further five studies (4%) go beyond discussion to implement or test wearable-informed HVAC control, demonstrating energy savings in experimental or simulated settings. The remaining 32 studies (27%) do not address energy implications. This distribution suggests that while the research community broadly recognizes the potential of wearable-based comfort models to inform energy-efficient building operations, the translation from sensing and prediction to actual building system integration remains largely aspirational, a gap explored in Section 6 of this review.

Finally, several partially overlapping constructs appear throughout the corpus within the thermal sensation/comfort/preference/personal comfort model and are inconsistently used in the literature. Therefore, this review adopts the following operational definitions, consistent with the ASHRAE 55-2023 framework, in order to normalize the analysis: (i) thermal sensation (typical scale: 7-point ASHRAE TSV: −3, cold to +3, hot) represents the conscious perception of the thermal environment; (ii) thermal comfort (typical scale: binary or Likert) represents the overall acceptability of the thermal environment; (iii) thermal preference (typical scale: three-class: cooler/no change/warmer) represents as the desired direction of change in conditions; and (iv) personal thermal comfort model represents any data-driven model predicting one of the above constructs from individual physiological or environmental inputs.

**Figure 11:**
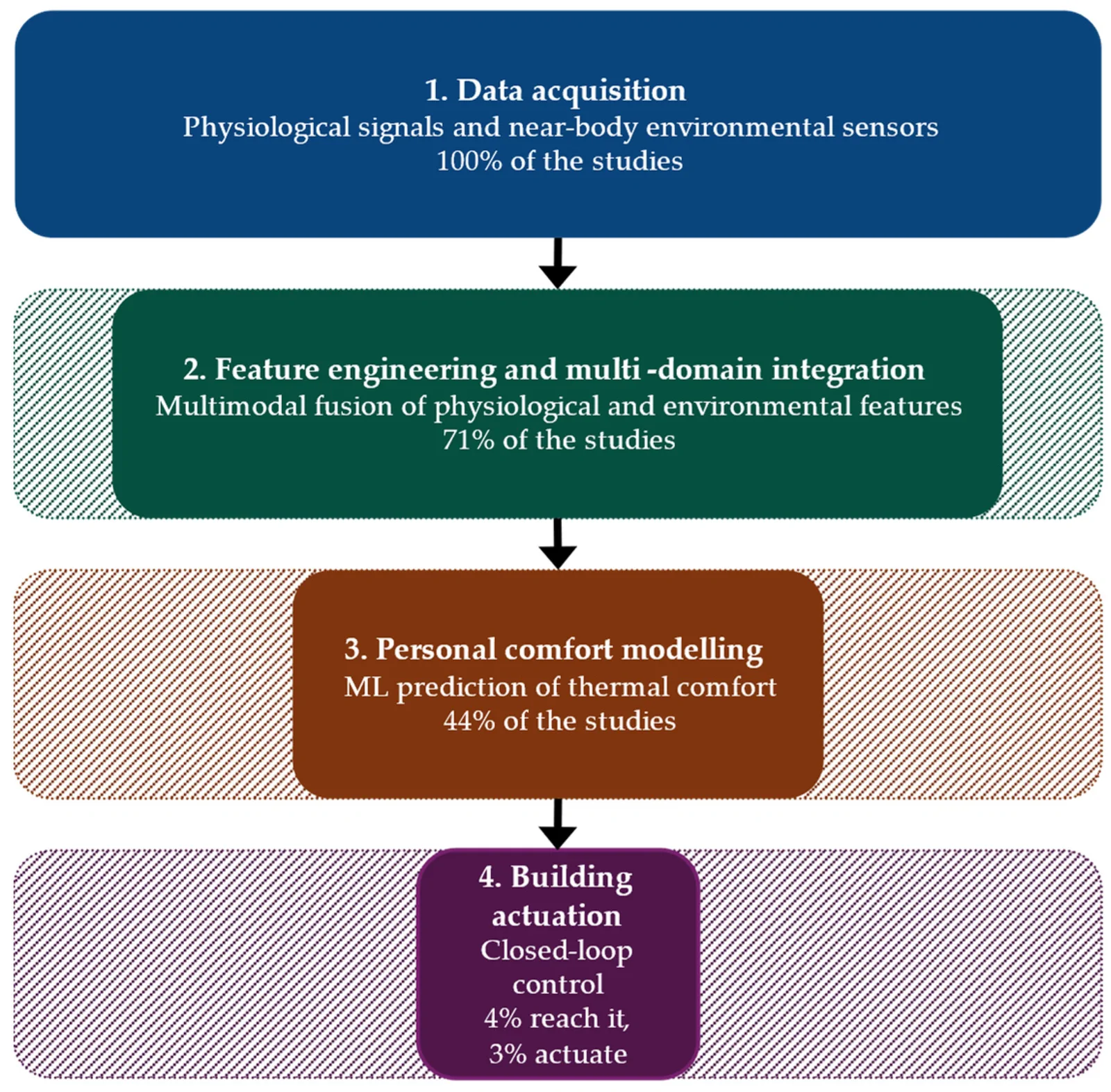
Conceptual framework: the wearable thermal comfort research pipeline.

To synthesize the overview and to organize the analyses presented in the following sections, the corpus can be mapped onto a four-stage conceptual pipeline that traces wearable thermal comfort research from raw signal acquisition to building actuation, reported in Figure 11.

In Figure 11, the bar width is proportional to corpus coverage at each stage, visualizing the implementation gap between sensing/modeling capability and actuation deployment. The first stage encompasses physiological and near-body environmental data acquisition; the second covers feature engineering and the integration of multimodal inputs; the third addresses personal comfort modelling, where physiological and contextual features are mapped to thermal sensation, preference, or comfort; the fourth concerns the deployment of these models within building systems for closed-loop control of HVAC or personal comfort systems. Every study contributes to the sensing stage; 83 (71%) integrate physiological and environmental signals, 52 (44%) develop machine learning models, and only 5 (4%) reach the building-actuation stage, of which 3 implement closed-loop control of a building system or a personal comfort device [24,65,66,67,68]. This narrowing organizes the analysis that follows: Section 4 examines Stage 1, Section 5 covers Stages 2 and 3, and Section 6 addresses Stage 4.

## 4. Wearable Sensing Technologies for Thermal Comfort

This section examines Stage 1 of the pipeline framework introduced in Figure 11, providing a structured examination of the sensing technologies deployed across the corpus, organized by signal domain. For each domain, the underlying physiological rationale, the devices and body locations employed, and the practical constraints affecting data quality are discussed. Table 2 synthesizes the hardware landscape, mapping device categories to the signals they capture, their typical body placement, and the associated strengths and limitations for thermal comfort research. This section directly addresses the gap identified in prior reviews [16,24,25], which cataloged devices without systematically connecting hardware characteristics to their implications for comfort modeling.

### 4.1. Skin Temperature Sensing

Skin temperature (SKT) is the most widely measured physiological signal in the corpus, appearing in 77 studies (66%). SKT measurement in the corpus follows two distinct paradigms. The first relies on miniature, single-purpose temperature loggers, predominantly the Maxim iButton family [51] (DS1922L, DS1923, Hygrochron), which are attached directly to the skin at multiple anatomical sites using medical tape or elastic bands. The iButton appears in 22 studies, of which 19 use it for multi-point skin temperature mapping, two for near-body environmental monitoring [69,70], and one for near-body temperature logging in children’s microenvironment [71]. The iButton remains the de facto standard for multi-point skin temperature mapping, owing to its small form factor, cost, logging autonomy and accuracy. Common multi-site protocols involve 4 to 14 measurement points distributed across the wrist, forehead, chest, upper arm, thigh, calf, and hand.

The second paradigm uses integrated wearable platforms where SKT is one of several co-acquired signals. The Empatica E4 wristband (18 studies) measures wrist skin temperature via an infrared thermopile alongside EDA, BVP, and accelerometry. Consumer smartwatches (Apple Watch—eight studies, Samsung Galaxy Watch—four studies, Fitbit—seven studies) also incorporate wrist temperature sensors, though their specifications are less transparent and their sampling rates are typically lower. At the head, the Muse EEG headband appears in seven studies, of which five capture forehead skin temperature as a secondary channel alongside EEG, and two record EEG only. The wrist is the dominant measurement site for integrated devices (70 studies, 60%), driven by the commercial smartwatch form factor. However, wrist skin temperature is known to be influenced by hand activity, vascular anatomy (proximity to the radial artery), contact with surfaces, and ambient air movement. These factors can introduce noise not representative of the core thermal state. Multi-site configurations mitigate this limitation: studies employing ≥4 measurement points consistently report stronger correlations between mean skin temperature and thermal sensation votes than single-wrist measurements [47,48,50,72,73,74,75,76,77,78].

Key challenges in SKT sensing include sensor attachment compliance (particularly in field studies lasting more than a few hours), thermal bridging effects (where the sensor mass itself alters local skin temperature), and the distinction between contact-based and infrared measurement methods. Contact thermistors and thermocouples (used in seven studies with custom prototypes) offer faster response times (<1 s) but require firm skin contact. Infrared sensors (as in the Empatica E4) are less intrusive but measure a weighted average of skin and ambient temperatures, which depends on the sensor-skin distance and emissivity assumptions.

### 4.2. Cardiovascular Sensing

Heart rate (HR) and its variability (HRV) appear in 69 and 33 studies, respectively, making cardiovascular metrics (including also PPG, BVP, IBI, and ECG) the most common physiological domain in the corpus (89 studies, 76%). The physiological rationale is well established: thermoregulatory demands increase cardiac output (raising HR) while shifting autonomic balance toward sympathetic dominance (reducing HRV), both responses that precede and accompany changes in subjective thermal sensation.

Two measurement approaches are most used across the corpus:

- Photoplethysmography (PPG) is the more common approach, used in wrist-worn devices such as the Empatica E4, Apple Watch, Samsung Galaxy Watch, and Fitbit. PPG detects blood volume changes through optical sensing (typically a green LED at 520–530 nm), from which HR and, with appropriate algorithms, HRV can be derived. PPG is convenient and non-intrusive but susceptible to motion artifacts, particularly during walking or physical activity, a significant limitation for field studies and outdoor comfort assessment [61,79,80,81,82,83].
- Electrocardiography (ECG) provides clinical-grade R-R interval measurement. 10 studies explicitly analyze ECG waveforms, while an additional 8 derive HR or HRV from ECG-based chest straps without reporting the raw ECG signal. These studies typically employ chest straps (Polar H7/H10; Zephyr BioHarness) or research-grade systems (BIOPAC; biosignalsplux; PowerLab). ECG enables more accurate HRV analysis, including frequency-domain metrics (LF/HF ratio) that are physiologically informative for autonomic assessment, but requires chest electrode placement that is more intrusive than wrist-worn alternatives [44,49,68,81,84,85,86,87,88,89].

Among the 33 studies measuring HRV, the most commonly extracted features are time-domain metrics (SDNN, RMSSD, pNN50) and frequency-domain metrics (LF power, HF power, LF/HF ratio), as observed in studies that report explicit HRV features [62,63,68,80,86,90,91,92,93,94,95]. The LF/HF ratio, reflecting sympathovagal balance, has been proposed as a particularly relevant indicator of thermal stress, as heat exposure shifts autonomic tone toward sympathetic dominance. However, HRV analysis requires stationary recording periods (typically ≥5 min for frequency-domain analysis), which conflicts with the continuous, ambulatory monitoring paradigm central to wearable comfort assessment. Several studies [62,96,97] address this tension by computing HRV features over sliding windows of 2–5 min, accepting reduced frequency resolution in exchange for temporal continuity.

### 4.3. Electrodermal Activity

Electrodermal activity (EDA), also referred to as galvanic skin response (GSR) or skin conductance (SC), appears in 30 studies (26%). EDA measures the electrical conductance of the skin, which varies with eccrine sweat gland activity under sympathetic nervous system control. Because sweat onset is one of the earliest thermoregulatory responses to heat exposure, often preceding conscious perception of thermal discomfort, EDA provides a potentially anticipatory signal for comfort transitions [80,91]. The Empatica platform is the most common device for EDA measurement in the corpus, with the E4 wristband appearing in 15 studies and its successors (EmbracePlus/Embrace 2) in 2 more, totaling 17 of the 30 EDA studies. These devices position two stainless steel electrodes on the ventral wrist and sample at 4 Hz. The remaining 13 studies employ research-grade systems such as BIOPAC (2 studies) and PowerLab (1 study), consumer wearables including Microsoft Band (2 studies) and ErgoLAB (2 studies), the EmotiBit platform (1 study), custom wireless EDA sensors (3 studies), and standalone GSR sensors (2 studies). Among these non-Empatica studies, six measure EDA at the fingers or hand, where the higher density of eccrine glands on the palmar surface generally yields higher signal quality, albeit at the cost of greater intrusiveness compared to wrist-based measurement [43,95,98,99,100,101]. Practical challenges with EDA measurement include high inter-individual variability in baseline conductance, sensitivity to electrode contact pressure, signal contamination from physical activity, and the relatively slow response dynamics of the tonic (skin conductance level) component compared to the phasic (skin conductance response) component. At least six studies in the corpus decompose EDA into tonic and phasic components [45,79,80,91,102,103], extracting features such as tonic standard deviation, tonic percentiles, SCR frequency and amplitude, and electrodermal level/response (EDL/EDR). These decomposed features may be more informative for comfort classification than raw conductance values: for example, the studies in [80,91] identify tonic EDA features among the top-ranked predictors in their feature importance analyses.

### 4.4. Electroencephalography

Electroencephalography (EEG) appears in 14 studies (12%), making it the most common neurophysiological signal in the corpus. The physiological rationale connects thermal comfort to cortical activity: thermal discomfort is associated with changes in attentional resource allocation, arousal, and affective processing that manifest as shifts in EEG power spectra, particularly in the alpha (8–13 Hz), beta (13–30 Hz), and theta (4–8 Hz) bands.

The Muse headband [55] (InteraXon) is the most frequently used EEG device in the corpus (seven studies), providing four-channel dry-electrode EEG at frontal (AF7 and AF8) and temporal (TP9 and TP10) positions. The remaining seven studies employ higher-specification systems spanning a wide range of channel counts: the Emotiv EPOC X [104] with 14 channels, the DSI-24 [105] with 19 active channels, the B-Alert x24 [106], the Neuracle [107] eight-lead wireless system, the NeXus-4 [108], and a Biosignals Plux EEG sensor [109] with two frontal channels. Channel counts in these non-Muse studies thus range from 2 to 24, and electrode technologies vary from dry sensors (DSI-24) to saline-soaked felt pads (Emotiv EPOC X) and conventional gel-based electrodes (BIOPAC, Biosignals Plux). The trade-off between systems is evident: consumer-grade headbands like the Muse enable practical deployment in field and office settings but capture only a limited spatial representation of cortical activity. In contrast, higher-channel systems provide richer spatial and spectral information but impose constraints on participant mobility and comfort that are less compatible with naturalistic monitoring.

EEG-based thermal comfort studies typically extract band power features across multiple frequency bands (alpha, beta, gamma, theta, and delta) along with band power ratios (e.g., alpha/beta ratio, in [46,110]; PSD(d)/[PSD(a) + PSD(b)] in [111]), temporal/frontal asymmetry indices [79,91,110], and spectral features such as spectral edge frequency [112]. Some studies extract more complex descriptors, including approximate entropy [102] and sensorimotor rhythm power [112]. Notably, 5 of the 14 EEG studies use EEG as the sole physiological signal, while the remaining nine combine EEG with other wearable signals such as SKT, HR/HRV, and EDA. A notable methodological concern is that EEG is highly sensitive to muscle artifacts (EMG contamination from jaw clenching, eye movements, and head motion), which are prevalent in non-laboratory settings and require sophisticated artifact rejection or correction algorithms (e.g., ICA decomposition) that are not consistently reported across the corpus [46,68,76,79,80,84,91,96,102,110,111,112,113,114].

### 4.5. Near-Body Environmental Sensing

While physiological signals capture the body’s response to thermal conditions, near-body environmental sensing measures the thermal stimulus itself at the occupant’s immediate location, capturing microenvironmental variations that fixed building sensors may miss. A total of 34 studies (29%) employ near-body environmental sensors, which can be grouped into four categories. The first and most common category uses iButton Hygrochron loggers [51] for ambient air temperature and relative humidity rather than skin temperature (10 studies). These are typically clipped to clothing or placed in pockets to measure microenvironmental conditions at the body surface. A second category relies on standalone portable environmental instruments (11 studies), including Kestrel portable weather meters [115], HOBO data loggers [116], UbiBot sensor nodes [117], and hybrid station configurations with globe thermometers for mean radiant temperature, pyranometers for solar radiation, and wind sensors. Third, wearable-embedded environmental sensors appear in 7 studies where compact sensor modules, such as BME280/BMP280 [118], DHT22 [119], and SHT series [120], are integrated into custom IoT wearable platforms alongside physiological sensors, measuring air temperature, relative humidity, and, in some cases, barometric pressure, CO_2_, TVOCs, and illuminance. Finally, a fourth category uses ambient sensors embedded in consumer devices (six studies), such as the Apple Watch’s sound-level sensor and smartphone-based temperature and humidity readings. The integration of near-body environmental data with physiological signals is characteristic of the multimodal approach that defines most of the corpus: 83 studies (71%) are classified as multimodal in their primary sensing domain. However, a methodological tension exists: near-body sensors measure conditions at the instrument location, which may differ from the thermal stimulus experienced by the skin surface where physiological sensors are placed. This spatial mismatch (e.g., between air temperature at belt height and skin temperature at the wrist) is rarely discussed or quantified in the reviewed literature, though it can introduce systematic bias in the feature matrices used for comfort modeling.

### 4.6. Less Common Signals and Emerging Trends Toward Multi-Signal Integration

Several physiological signals appear in fewer than 10 studies each but represent potentially valuable additions to the sensing repertoire. Blood oxygen saturation (SpO_2_; 11 studies) is increasingly available on consumer smartwatches and may serve as an indirect indicator of cardiovascular strain during thermal stress. Metabolic rate estimation (6 studies) addresses one of the most uncertain inputs to conventional comfort models (PMV), with approaches ranging from indirect calorimetry [64] to activity-based estimation using accelerometry data [121,122]. Blood pressure (seven studies) captures cardiovascular responses complementary to HR/HRV, though continuous non-invasive BP measurement remains technically challenging with wearable devices. More exploratory signals include respiratory rate and airflow (six studies), sweat rate and skin humidity (four studies), heat flux (two studies), salivary cortisol (one study), continuous blood glucose via a FreeStyle Libre 2 monitor [123] (one study), and facial EMG (one study). While individually rare in the corpus, these signals collectively illustrate the expanding frontier of wearable physiological monitoring for comfort research and the potential for multimodal fusion approaches that combine established signals (SKT, HR, and EDA) with emerging indicators to improve prediction robustness.

A clear trend in the corpus is the increasing adoption of multi-signal sensing configurations. Among the 117 studies, 6 employ environmental sensing only, without physiological signals [17,69,70,71,124,125], 19 capture a single physiological signal, 31 capture two signals, 42 capture three or four signals, and 19 capture five or more. The convergence of multiple sensors enables this progression toward richer signal spaces into single wearable platforms (e.g., the Empatica E4 [38] alone provides SKT, EDA, BVP/HR, and accelerometry), while research-grade systems such as the BIOPAC MP160 [106] and Biosignals Plux [109] can capture even broader signal sets. Li et al. [86] is the most signal-rich study in the corpus, simultaneously records SKT, HR, HRV, EDA, blood pressure, SpO_2_, and ECG, while Kim et al. [94] is the only study in the corpus to combine endocrine (salivary cortisol) and metabolic (blood glucose) biomarkers with conventional physiological signals, framing thermal comfort assessment within a broader psychophysiological stress paradigm. However, multi-signal configurations increase participant burden (more devices, more body sites, more data management), amplify data quality issues (synchronization across devices, differential sampling rates, missing data), and risk model overfitting when the number of features exceeds the number of training samples [86]. The most informative configurations balance signal diversity against practical viability, a balance that the next section examines through the lens of modeling performance.

**Table 2:** Summary of the main characteristics of common experimental devices.

Category	Representative Models	Number of Studies	Signals Captured	Body Location	Samp. Rate	Strengths	Limitations
Temperature loggers	iButton DS1922L/DS1923/DS1925, Hygrochron, Thermochron	22 [7,47,48,50,63,65,69,70,71,72,73,74,75,76,77,78,97,126,127,128,129,130]	SKT (single-channel); near-body T/RH (Hygrochron models)	Multi-site (4–14 points): wrist, forehead, chest, arm, thigh, calf, hand; or belt/pocket (environmental)	0.017–0.2 Hz (5–60 s intervals)	Miniature (17 mm); low cost (~$30–50); autonomous logging; multi-point mean SKT; field-proven	No real-time streaming; manual data retrieval; thermal bridging; limited temporal resolution
Multimodal wristband (research)	Empatica E4, EmbracePlus, Embrace 2	18 [19,45,46,61,67,79,80,82,91,92,94,102,131,132,133,134,135,136]	SKT (IR thermopile); EDA (4 Hz); BVP/HR (64 Hz); 3-axis ACC (32 Hz)	Wrist (ventral)	4–64 Hz (signal-dependent)	Multi-signal single device; research-validated; real-time BLE streaming; de facto standard for cross-study comparison	Wrist-only (single SKT site); motion artifacts on BVP/EDA; high cost (~$1700); E4 discontinued
Consumer smartwatch, smartband	Apple Watch, Samsung Galaxy Watch, Fitbit, Garmin, Huawei, Oura Ring, others	24 [7,41,62,63,73,90,93,96,97,122,128,135,137,138,139,140,141,142,143,144,145,146,147,148]	HR (PPG); SpO_2_; wrist temperature; ACC; step count; sound level (Apple Watch)	Wrist; finger (Oura Ring)	~0.1–1 Hz (model-dependent); typically, 1 min HR averages	Ubiquitous; high user acceptance; long battery life; low cost ($200–400); large-scale deployment potential	Opaque algorithms; coarse temporal resolution; limited raw data access; no EDA (most models); not research-validated
Chest strap (HR/ECG)	Polar H7/H10, Zephyr BioHarness 3.0	8 [47,48,50,64,76,77,85,129]	ECG (250–1000 Hz); HR; HRV (R-R intervals); respiratory rate; ACC	Chest (sternum band)	250–1000 Hz	Clinical-grade R-R intervals; accurate HRV frequency-domain analysis; robust to motion	Intrusive chest placement; low compliance in field studies; single body site; no SKT or EDA
EEG headband (consumer)	Muse, Muse 2, Muse S (InteraXon)	7 [46,79,80,84,91,102,110]	EEG (4-ch: AF7, AF8, TP9, TP10); forehead SKT (5 studies); PPG (Muse 2+)	Head (frontal/temporal)	256 Hz	Dry electrodes; consumer-grade wearability; captures cortical arousal/attention; simultaneous forehead SKT	Only 4 EEG channels; high EMG artifact susceptibility; limited spatial resolution; conspicuous head-mounted form
Research-grade EEG	Emotiv EPOC X, DSI-24, B-Alert X24, Neuracle, NeXus-4, Biosignals Plux EEG	7 [68,76,96,111,112,113,114]	EEG (2–24 channels); some include ACC	Scalp (cap or headset)	128–512 Hz	Higher channel counts (2–24); richer spectral/spatial analysis; clinical validation	Intrusive; gel or saline application (some models); restricted mobility; impractical for field deployment
Research-grade physiology	BIOPAC MP160/MP36R, biosignalsplux/PLUX, ErgoLAB, PowerLab	13 [43,44,68,81,86,87,88,89,92,98,101,149,150]	ECG; EMG; EDA; respiration; SKT (multi-channel)	Multiple sites (study-dependent)	500–2000 Hz	Gold-standard signal quality; multi-channel simultaneous acquisition; full raw data access; customizable protocols	Tethered (wired); lab-only; expensive ($5000+); requires trained operator; limited ecological validity
Custom, prototype IoT	Arduino/ESP32-based platforms; custom PCBs with BME280, DHT22, SHT, MLX90614, NTC thermistors	23 [17,24,40,42,85,95,99,103,125,151,152,153,154,155,156,157,158,159,160,161,162,163,164]	Variable: SKT (IR/thermistor); T/RH/P (near-body env); CO_2_; PM2.5; noise; illuminance	Variable: wrist, face mask, helmet, belt, desk-mounted	0.1–10 Hz (variable)	Fully customizable; low per-unit cost; combines physio + env sensing; open-source firmware; tailored to research needs	Not commercially validated; variable build quality; limited inter-study comparability; no standardized calibration
Portable weather station	Kestrel 5400/Drop D2, HOBO data loggers, HD33MT.4	4 [139,145,146,165]	Air temperature; RH; air velocity; globe temperature; barometric pressure	Handheld or belt-mounted	1–60 s intervals	Multi-parameter environmental profiling; established calibration protocols	Environmental only (no physio); bulky for continuous carry; requires manual positioning

## 5. Personal Comfort Modeling

This section examines Stage 2 and 3 of the pipeline framework (Figure 11), exploring the modeling dimension of the corpus, connecting wearable data to comfort prediction. The analysis follows the three-stage pipeline identified by Gao and Ghahramani [20] (feature engineering, model selection, and validation), while extending it with a cross-study performance comparison that prior reviews have not provided in structured form. Of the 117 studies in the corpus, 52 (44%) employ machine learning (ML) models, summarized in Table 3. This section focuses on these studies while drawing on the statistical analyses in the remaining 65 to provide context on feature relevance and physiological associations.

### 5.1. Output Variables and Comfort Scales

The choice of prediction target varies across the corpus and reflects differing conceptual framings of what wearable sensing should predict. Because many studies predict multiple outputs simultaneously (e.g., Cen et al. [50] predict TSV, thermal preference, and air velocity preference), the counts below reflect the number of studies in which each target appears, with overlap across categories. The thermal sensation vote (TSV) is the most common output variable, appearing in 25 of 52 ML studies (48%). Thermal comfort and related constructs (comfort/discomfort, thermal satisfaction, thermal state) appear in 14 studies (27%). Thermal preference (TP) appears in nine studies (17%). Three studies predict the PMV index directly from physiological inputs, while 11 use PMV predictions as a comparison benchmark against their wearable-based models (see Table 3). Less common targets include metabolic rate estimation (two studies), clothing insulation prediction (one study), binary heat event detection (one study), and core body temperature (one study).

This heterogeneity presents a fundamental challenge for cross-study comparison: a model predicting three-class thermal preference (want cooler/no change/want warmer) solves a structurally different problem from one predicting seven-point thermal sensation (cold to hot). The number of output classes varies accordingly: 16 ML studies use a three-class formulation as their primary output, 12 employ binary outputs, 10 retain the full seven-point ASHRAE scale or wider, 5 use five-class scales, and the remaining studies employ nine-point (3 studies) or continuous outputs (6 studies). This inconsistency, rarely acknowledged in individual studies, makes accuracy comparisons across the corpus inherently problematic, as baseline random-chance accuracy varies from 14% (seven-class) to 33% (three-class) to 50% (binary). Table 3 aggregates model performance by output category to enable more meaningful cross-study comparison within comparable prediction tasks.

### 5.2. Input Feature Definition

The input features used across ML studies span four broad categories: physiological signals, environmental parameters, personal/demographic attributes, and contextual variables. Among physiological features, skin temperature is the most frequently included (45 studies, 87% of ML studies), followed by heart rate (39, 75%), EDA/GSR/skin conductance (20, 38%), HRV-derived metrics (11, 21%), activity/accelerometry (6, 12%), and EEG (5, 10%). Environmental parameters drawn from near-body or fixed sensors (including air temperature, relative humidity, air velocity, globe temperature, CO_2_, and illuminance) appear as model inputs in 34 studies (65%). Personal attributes (age, sex, BMI, weight, and height) are included in 15 studies (29%), clothing insulation estimates in 8 (15%), and metabolic rate estimates in 7 (13%).

Most studies extract statistical aggregates from physiological time-series: mean, standard deviation, minimum, maximum, range, and percentiles computed over fixed-duration windows. Temporal derivatives and rates of change capture the dynamics of thermoregulatory responses, which may be more predictive of comfort transitions than absolute values. Frequency-domain features, particularly for HRV (LF/HF power ratio, total spectral power; e.g., [62,63,68,80,86,90,91,92,93,94,95] and EEG (band power ratios; e.g., [46,79,80,91,102,110,111,112]), appear in a subset of studies and require stationary recording segments that are difficult to guarantee in ambulatory settings. A smaller number of studies compute difference features, such as the gradient between skin and ambient temperatures (e.g., [24,50,92]), which encode the thermal driving force experienced by the occupant. A notable gap is the near absence of advanced signal processing methods: none of the 52 ML studies employ wavelet decomposition, empirical mode decomposition, or deep feature extraction (e.g., autoencoders) for physiological signal representation. To assess whether combining physiological and environmental features improves predictions, the corpus was stratified by input feature type. Studies using combined physiological and environmental features (23 studies) achieve a median accuracy of 90%, compared to 86% for physiological-only models (15 studies) and 92% for environmental-only models (3 studies). The higher median accuracy of environmental-only models likely reflects the small sample size (three studies; [62,84,121]) and their use of rich sensor arrays in controlled settings. The consistent four-percentage-point improvement from physiological-only to combined models is more informative, confirming that environmental context enhances physiological-based predictions. This finding supports the multimodal approach adopted by 71% of the corpus.

### 5.3. Algorithm Selection and Performance

The ML studies in the corpus employ a diverse range of algorithms, often benchmarking multiple approaches on the same dataset. At the study level, the most frequently adopted are Support Vector Machines (31 studies, 60%) and Random Forest (26, 50%), followed by Artificial Neural Networks and related architectures (ANN/MLP/DNN; 19, 37%), k-nearest neighbors (16, 31%), regression-based methods including logistic, linear, and ridge regression (15, 29%), decision trees (11, 21%), gradient boosting methods (XGBoost, LightGBM, CatBoost; 9, 17%), deep learning architectures (CNN, LSTM, and GRU; 8, 15%), Naive Bayes (7, 13%), ensemble boosting methods such as AdaBoost (3, 6%), and clustering approaches (1, 2%). Table 3 disaggregates the most common methods by output category, revealing that SVM and RF dominate across all prediction targets.

Among the 41 studies reporting classification accuracy or F1 score, the overall distribution ranges from 67% to 99%, with a median of 90% and a mean of 88%. However, these aggregate values should be interpreted with caution: the apparent comparability of median accuracies across algorithms masks substantial within-algorithm variance that is driven more by data characteristics (number of classes, sample size, signal quality) than by algorithm choice. As Table 3 shows, the output category is a stronger predictor of reported accuracy than the algorithm family: thermal preference models achieve a median of 79%, comfort/discomfort binary models 86%, and TSV 3-class models 90%, regardless of the algorithm used.

The eight studies employing deep learning architectures [62,74,100,130,133,140,141,166] represent the emerging frontier of comfort modeling. CNNs are typically applied to extract spatial features from multi-channel physiological or environmental data, while LSTMs and GRUs model temporal dependencies in physiological time series. The analysis reported in Park & Park [133] achieves 95% accuracy with a CNN-SVM ensemble using transfer learning from a pre-trained physiological dataset (three participants). In comparison, the one in Liu et al. [166] reports 98% with a hybrid RF-BPNN-LSTM architecture (16 participants). Despite these promising individual results, deep learning approaches have not consistently outperformed classical ensemble methods in the corpus. The likely explanation is data scarcity: with a median sample size of 15 participants across the 52 ML studies (range 1–103; see Table 3), most datasets are too small to exploit the representational capacity of deep architectures. The few deep learning studies reporting strong performance either use data augmentation, pre-training on external datasets, or operate on larger datasets (e.g., Li et al. [74], N = 51). This observation suggests that deep learning’s potential for thermal comfort modeling may be constrained less by algorithmic limitations than by the data-collection paradigms currently prevalent in the field.

### 5.4. Validation Strategies

The choice of validation strategy profoundly affects reported performance and determines whether a model can be expected to generalize to new individuals and environments. Among the 52 ML studies, k-fold cross-validation is the most common approach (28 studies, 54%), followed by hold-out train/test splits (12, 23%), and leave-one-subject-out cross-validation (LOSOCV; 8, 15%). The remaining studies employ clustering validation (one study), PMV comparison (one study), or other within-subject designs (two studies).

The performance difference across validation strategies is the most consequential finding of this cross-study analysis and is detailed in Table 4. Studies using k-fold CV report a median accuracy of 90% (IQR: 79–94%; 25 studies, range 67–99%), hold-out split studies report 90% (IQR: 86–96%; 8 studies, range 73–99%), and LOSOCV studies report 85% (IQR: 78–88%; 6 studies, range 71–92%). These figures should be interpreted with caution: they aggregate heterogeneous studies with different numbers of output classes, class distributions, and sample sizes, and are not directly comparable in a statistical sense. The five-percentage-point gap between k-fold CV and LOSOCV is likely due to the accuracy gained from training on the same individuals who appear in the test set. By training on all subjects except one and testing on the held-out individual, LOSOCV evaluates inter-individual generalization: the ability of a model trained on a population to predict a new person’s comfort. This is a fundamentally harder problem than intra-individual prediction (where training and test data come from the same person, as in standard k-fold CV). However, it is also the more practically relevant one for building systems that must serve occupants without prior calibration. It is important to note that within-subject k-fold CV is not inherently inappropriate: for personalization models, where a system is explicitly calibrated on a specific user’s prior data before deployment, within-subject validation is the correct evaluation strategy. The criticism applies specifically to studies claiming population-level generalizability while using a validation design that leaks individual identity between training and test sets, thereby inflating the apparent performance of what is effectively a subject-specific model.

As Table 4 highlights, hold-out split studies report a median accuracy of 90% (IQR: 86–96%) but with lower statistical robustness than cross-validation approaches, as results depend on a single random partition. The observed gradient from LOSOCV through k-fold CV to hold-out is consistent with the expected effect of data leakage across splits. However, it should be interpreted as indicative rather than definitive, given the small number of LOSOCV studies (n = 6) and the heterogeneity of tasks and datasets involved. Practically, this suggests that models calibrated on a training population may perform several percentage points worse on new occupants unless adaptive or transfer learning mechanisms are incorporated, which is a direction explored by only three studies in the current corpus [133,141,167]. Table 5 provides a brief decision framework for selecting the appropriate validation strategy based on the intended modeling objective.

### 5.5. Transfer Learning and Comparison Against Conventional Comfort Indices

Transfer learning and domain adaptation represent arguably the most important and most underexplored frontier in wearable-based comfort modeling. Only 3 studies [133,141,167] in the corpus explicitly address generalization across individuals or contexts. Park & Park [133] propose a CNN-SVM ensemble with transfer learning, achieving 95% accuracy by pre-training on a large physiological dataset and fine-tuning on a small comfort-labeled dataset (three participants). Yang et al. [141] develop SMAT (Selective Multi-source Adaptation for Thermal comfort), a domain adaptation method that selectively transfers knowledge from multiple source domains. Natarajan & Laftchiev [167] use ridge regression with transfer active learning, reducing the amount of labeled data needed from a new user. The scarcity of transfer learning studies is a critical gap. In practice, any wearable-based comfort system deployed in a building must handle new occupants without requiring days of labeled calibration data. The evidence base of three studies from three research groups is far too thin to support confident deployment recommendations. This contrasts with adjacent fields such as activity recognition and emotion detection, where transfer learning across individuals is an active and mature research area. Eleven studies in the corpus directly compare the performance of their wearable-based models against PMV on the same dataset (see Table 3, last column). Among those reporting classification accuracy, the wearable-based models are associated with higher reported accuracy than PMV, with improvements typically ranging from 10 to 20 percentage points across the studies that report this comparison. Two studies predict the PMV index itself from wearable inputs [156,157], achieving near-perfect agreement (R^2^ = 0.999, RMSE = 0.023) by estimating metabolic rate from physiological data rather than assuming fixed values, demonstrating that wearables can improve PMV predictions even within the conventional framework. However, while 11 studies include PMV comparison, this still represents only 21% of the 52 ML studies. Without systematic benchmarking against the established standard, it is difficult to assess whether the added complexity, cost, and intrusiveness of wearable sensing are justified by the performance gains they deliver. Moreover, no study reports a comparison against the adaptive comfort model of ASHRAE 55 [4] or EN 16798-1 [168]. For the field studies, as PMV was derived for steady-state, mechanically conditioned environments and is known to mis-predict sensation in naturally ventilated and mixed-mode buildings [9], benchmarking a wearable-based model only against PMV can overstate the added value of physiological sensing. For field studies, the adaptive model, computed from the prevailing mean outdoor temperature, may be the more appropriate reference to be reported alongside PMV. Future studies should routinely include PMV/adaptive model predictions as a baseline, computed from the same environmental measurements used in the wearable-based model. This methodological practice would greatly strengthen the evidence base for wearable-based comfort approaches.

### 5.6. Synthesis on Personal Comfort Models

Drawing together the analyses in Section 5, and as summarized in Table 3, Table 4 and Table 6, the observational evidence suggests that model performance is strongly influenced by five principal factors:

- The formulation of the output variable is a major determinant of reported accuracy. As Table 3 shows, thermal preference models achieve a median accuracy of 79%, comfort/discomfort binary models 86%, and TSV 3-class models 90%, reflecting both the differing difficulty of these prediction tasks and the theoretical baseline shift from 50% (binary) to 33% (3-class) random chance. Accuracies obtained on different targets are therefore not commensurable.
- The validation strategy is associated with an indicative difference of approximately 5 percentage points between k-fold CV (median 90%, IQR 79–94%) and LOSOCV (median 85%, IQR 78–88%) (Table 4), a magnitude comparable to or larger than the differences between most algorithm families. This underscores that the evaluation of a model matters at least as much as which algorithm is chosen. These comparisons should be read as exploratory trends across heterogeneous studies rather than precise statistical estimates. The mechanism is identity leakage [169]. Physiological signals carry a persistent individual signature, so when windows drawn from the same participant fall in both the training and the test folds, a classifier can obtain part of its apparent accuracy by recognizing the subject rather than the thermal state. On this reading, the difference does not measure performance lost, but the share of the apparent performance that leakage had supplied.
- The input feature composition is associated with higher reported accuracy for combined physiological and environmental features over physiological signals alone (+4 percentage points median accuracy). However, this seems to be related to disambiguation rather than added information: an elevated wrist skin temperature is consistent with a warm environment, with recent physical activity, and with an affective response (Section 4.1), and concurrent measurement of the near-body environment narrows which of these produced it. This predicts a benefit that is largest where the physiological signal is most ambiguous, and Table 6 is only partly consistent with it: combined inputs are associated with higher median accuracy for multi-class thermal sensation (91% against 88%) and for binary comfort classification (90% against 81%), but not for three-class thermal preference (78% against 80%).
- The algorithm choice has a surprisingly modest effect, with median accuracies clustering between 79% and 92% across all major algorithm families. Ensemble methods (Random Forest, gradient boosting) and SVM offer the most reliable performance with the least sensitivity to hyperparameter tuning, while deep learning approaches show high variance driven by data availability. As a practical consequence for deployment in building-control, where the predictive gain from additional model capacity is small, the operational costs of that capacity become decisive: interpretability, since a controller acting on an inferred physiological state must be easily auditable; computational and energy footprint, since inference on a battery-powered wearable or an edge gateway is constrained by a power budget; robustness to data loss, since models operating on low-dimensional aggregated features degrade gracefully when wireless physiological streams drop packets, whereas architectures depending on uninterrupted high-frequency input do not; and statistical appropriateness, since with a median of 15 participants and feature sets containing dozens of time-domain and frequency-domain descriptors, high-capacity models are structurally prone to overfitting [170].
- The sample size and data volume interact with algorithm choice: the median sample size of 15 participants (range 1–103) is adequate for classical methods. However, it constrains deep learning architectures, which require larger and more diverse datasets to realize their representational capacity.

These findings collectively suggest that the field’s primary research priorities should shift from algorithm optimization toward more fundamental challenges: standardized output variable definitions, rigorous LOSOCV validation as a minimum reporting standard, routine benchmarking against PMV, and, above all, larger, more diverse, and longitudinally collected datasets that can support robust generalization testing. Deep architectures are justified where the modeling target genuinely requires what they provide. Where the input is a small table of aggregated features, the simpler model is methodologically the appropriate choice to report as a baseline alongside any more complex alternative.

## 6. Integration with Building Systems and Energy Implications

The previous sections have established that wearable sensing can capture thermoregulatory responses with high fidelity (Section 4) and that machine learning models trained on these data achieve median prediction accuracies around 90% under within-subject validation (Section 5). This section examines Stage 4 of the pipeline framework (Figure 11), i.e., the integration of comfort models with building actuation, exploring the critical yet largely incomplete step of translating wearable-based comfort predictions into actionable building-system control strategies and quantifiable energy outcomes. This pathway, from sensing to prediction to control to energy impact, is where the reviewed literature is weakest, and where the gap between research promise and practical implementation is most evident.

Among the 117 studies in the corpus, the vast majority discuss HVAC or energy-saving implications of their findings at the conceptual level, typically including a paragraph in their conclusions noting that personalized comfort predictions could inform setpoint optimization, demand-driven ventilation, or occupant-centric HVAC scheduling, but without implementing, simulating, or quantifying these strategies. Only a small number of studies cross the implementation threshold by testing wearable-informed control in a physical or simulated building environment [24,65,66,67,68]. These represent the most practically advanced contributions in the corpus and deserve detailed examination. Table 7 classifies them by the depth of implementation actually reached, distinguishing laboratory from semi-controlled and field settings, recording what each study actuates, and noting whether an energy outcome was quantified.

Yoshikawa et al. [67] combined thermal camera-based facial temperature with Empatica E4 wristband data (HR, SKT, EDA), achieving higher comfort prediction accuracy than either modality alone. The system was tested in a real office environment with 15 participants, where real-time predictions informed HVAC adjustments.

Hu et al. [66] developed iTCM, an IoT-based thermal comfort management platform integrating Microsoft Band 2 wearable data with environmental sensors for 30 occupants. Their architecture (cloud-based data fusion, edge-level processing, and BMS-level actuation) represents one of the most complete system-level implementations in the corpus, demonstrating pervasive sensing for smart building comfort management.

Ren et al. [68] demonstrated activity-dependent localized heating using physiological feedback from ECG and EEG sensors (Biosignals Plux). Their key finding was that single-zone core heating was optimal during resting states. In contrast, multi-zone combined heating was needed during active states, achieving 75.6% energy reduction compared to conventional HVAC while maintaining equivalent comfort levels.

Cho et al. [24] developed a wristband-type wearable measuring three-point skin temperature that predicted thermal comfort with 93.9% accuracy using Random Forest. Critically, they demonstrated a proof-of-concept closed-loop HVAC system in which the wearable prediction directly adjusted the room temperature setpoint, resulting in improved comfort and reduced energy consumption in a single-occupant chamber. This study remains one of the few to close the full sensing/prediction/actuation loop.

Deng and Chen [65] built a smart HVAC control system for multi-occupant offices using physiological data from a commercial wristband (Hesvit S3: wrist skin temperature, skin relative humidity, heart rate) combined with environmental measurements. An ANN predicted individual thermal sensation votes, which were aggregated across 24 occupants to optimize the shared HVAC setpoint. The system improved group thermal satisfaction compared to conventional fixed-setpoint operation.

Of the three studies that actuate, only Deng and Chen [65] operate in an occupied building rather than a laboratory chamber, and only Ren et al. [68] report a quantified energy outcome, which refers to localized heating of a single occupant and therefore does not transfer to the zone-level control problem the other studies address. Thus, the gap in the corpus continues past the actuation stage: of 117 studies, five reach it, three actuate on a building or a personal comfort system, one does so in an occupied building, and one reports what that actuation cost or saved in energy terms.

The control strategies discussed or implemented across the corpus can be organized into four approaches. Personal comfort systems (PCSs) are the most frequently discussed integration pathway. These bypass centralized HVAC entirely, using localized devices (desk fans, personal heaters, heated/cooled chairs) whose operation is informed by wearable-derived comfort predictions. The PCS approach sidesteps the fundamental multi-occupant conflict inherent in shared HVAC zones: rather than finding a single setpoint that satisfies (or dissatisfies) everyone, it allows individual thermal microenvironments. Several studies show that coupling PCS with wearable monitoring enables raising cooling setpoints by 2–3 °C without a comfort penalty, a strategy that directly translates into substantial energy savings. However, PCS deployment is currently limited to sedentary office environments and does not address common building types (retail, healthcare, and education) where occupants are dynamic, and PCS placement is impractical.

Real-time feedback and adaptive control use wearable comfort predictions to modulate HVAC parameters continuously. This approach requires three capabilities that are each technically challenging: real-time data streaming from wearable devices, low-latency prediction inference, and a bidirectional interface between the comfort prediction system and the building management system (BMS). The implemented studies described above demonstrate that each component is technically feasible, but none has been deployed at scale beyond single rooms or small office sections.

Setpoint adjustment is the simplest integration approach: wearable-based comfort predictions are used to shift HVAC temperature setpoints toward the edges of the comfort zone, reducing conditioning energy while maintaining predicted satisfaction above an acceptable threshold. This strategy is attractive because it requires minimal BMS modification, as only the setpoint value changes. However, it cannot handle the temporal dynamics of occupant comfort or adapt to individual differences within shared zones.

Digital twin integration couples wearable-derived pedestrian comfort data with building/urban simulation models, enabling what-if analyses of design alternatives before physical implementation. This approach is represented in the corpus by Ignatius et al. [145], who used wearable data from an Apple Watch combined with urban environmental measurements to validate a digital twin model of pedestrian thermal comfort and walkability in Singapore.

A fundamental tension in wearable-based HVAC control is the mismatch between individual-level comfort prediction and zone-level HVAC operation. Most commercial HVAC systems operate at the zone level with a single thermostat controlling a space shared by multiple occupants. Wearable sensing produces individual-level predictions that may conflict: one person may be warm and uncomfortable while an adjacent person is comfortable, or even cool and uncomfortable. Only one study in the corpus [65] directly addresses multi-occupant aggregation, using weighted averaging of individual ANN predictions to determine a compromise setpoint for 24 occupants. The remaining studies either operate in single-occupant settings or defer the aggregation problem to future work. This gap is critical: real buildings are predominantly multi-occupant, and any practical deployment of wearable-based comfort control must solve the aggregation problem, either through democratic averaging (which guarantees some individual dissatisfaction), priority weighting (which raises equity questions), or spatial conditioning strategies that create individual microclimates within shared spaces.

The PCS approach offers a partial solution by decoupling individual comfort management from centralized HVAC, but, as noted above, this is feasible only in specific occupancy configurations. An alternative strategy, not yet explored in the reviewed corpus, would combine wearable-informed zone-level HVAC with PCS as a supplementary “fine-tuning” layer: the centralized system maintains conditions near the zone median preference, while PCS devices address individual deviations. This hybrid architecture could reconcile the tension between zone-level infrastructure and person-level sensing, but requires coordination algorithms that remain undeveloped.

The scarcity of quantified energy outcomes is perhaps the most significant gap in the corpus for the building engineering community. Only Ren et al. [68] report a specific energy savings figure: 75.6% energy reduction with localized heating compared to conventional HVAC. The remaining studies that discuss energy implications do so qualitatively, noting that personalized comfort models could reduce energy consumption, but offer no simulated or measured estimates.

The practical gap is consequential. Building operators and facilities managers make investment decisions based on quantifiable cost–benefit analyses. Without evidence that wearable-based comfort systems reduce energy consumption by a specific, replicable amount, and that this reduction justifies the cost of deploying and maintaining wearable sensing infrastructure, adoption will remain limited to research demonstrations. Future studies should include building energy simulation (e.g., EnergyPlus or TRNSYS) to estimate the energy impact of their comfort prediction strategies under realistic operating conditions, occupancy patterns, and climate scenarios.

Beyond the algorithmic and control challenges, several practical barriers limit the integration of wearable sensing with building systems. Sustained wearable use requires occupant willingness; while consumer smartwatches enjoy broad adoption for fitness and health monitoring, their use for building comfort management introduces new concerns, such as access to and use of physiological data. Studies mentioning IoT or smart building integration generally assume willing participation without addressing opt-in/opt-out mechanisms, privacy policies, or the degraded-mode operation that occurs when some occupants wear devices, and others do not.

Real-time wearable-to-BMS integration requires continuous data streaming, cloud or edge processing, and standardized communication protocols. The implemented studies that describe their system architecture (Cho et al. [24]; Deng & Chen [65]; Hu et al. [66]) each built custom middleware solutions, as there is no established standard or protocol for wearable-to-BMS data exchange analogous to BACnet or Modbus for traditional building sensors. The implemented studies involve 6 to 30 participants in single rooms or small office sections. Scaling to building-level deployment (hundreds of occupants, multiple zones, varying schedules) introduces challenges that are not simply quantitative extensions of the single-room case: data management scales linearly, but the multi-occupant aggregation problem scales combinatorially, and system reliability requirements increase nonlinearly with the number of connected devices.

The total cost of a wearable-based comfort system includes device procurement, replacement cycles (battery life and device obsolescence), data infrastructure, software maintenance, and occupant support, all of which are not offset unless energy savings and productivity gains exceed them. No study in the corpus provides a complete lifecycle cost analysis, making it impossible for practitioners to evaluate the economic viability of adoption.

Moreover, an emerging research direction connects outdoor wearable sensing to building system operation through the concept of thermal history and pre-conditioning. Twenty-three studies in the corpus cover outdoor environments (9 outdoor-only [17,43,97,101,125,145,165,171,172]; 14 both indoor and outdoor [41,47,48,69,73,87,124,126,129,142,143,144,146,147]), and several demonstrate that occupants’ thermal sensation upon entering a building is strongly influenced by their outdoor thermal exposure during the preceding minutes. This phenomenon, recognized in the adaptive comfort literature but rarely measured with physiological data, has direct implications for building operation: an occupant arriving from a hot outdoor environment may require more intensive initial cooling that tapers as physiological acclimatization occurs, while an occupant from a cool outdoor environment may tolerate or even prefer a warmer indoor setpoint. Wearable sensing is uniquely positioned to capture this transition, as the same device can record outdoor exposure (skin temperature and heart rate response to heat/cold) and indoor arrival conditions in a continuous data stream. However, the operational implications (e.g., preconditioning building zones based on wearable-sensed outdoor arrival patterns) remain theoretical and represent an untested yet potentially impactful application of continuous wearable monitoring.

### Synthesis: From Prediction to Practice

The analysis above reveals a field that has achieved substantial progress in the sensing and prediction stages of the wearable-to-building pipeline but has largely stalled at the integration stage. The five implemented studies [24,65,66,67,68] demonstrate that closed-loop, wearable-informed HVAC control is technically feasible and improves comfort while reducing or maintaining energy consumption. However, these remain isolated proof-of-concept demonstrations in controlled settings, not scalable building solutions.

Three priorities emerge for bridging this gap: (i) the research community must move beyond qualitative energy discussions toward quantified, simulation-supported energy impact assessments that building operators can compare with conventional system costs; (ii) the multi-occupant aggregation problem requires explicit algorithmic treatment (e.g., democratic averaging, priority-weighted optimization, or hybrid centralized-plus-PCS architectures) tested in realistic shared-zone configurations; (iii) practical deployment requires standardized wearable-to-BMS communication protocols, clear privacy frameworks for physiological data in workplaces, and degraded-mode operation strategies for buildings where wearable adoption is partial. These challenges are not insurmountable, but they require a shift in research focus from individual comfort prediction to system-level integration engineering, which has only begun to emerge in the literature.

## 7. Risk of Bias, Gaps, and Future Directions

**Figure 12:**
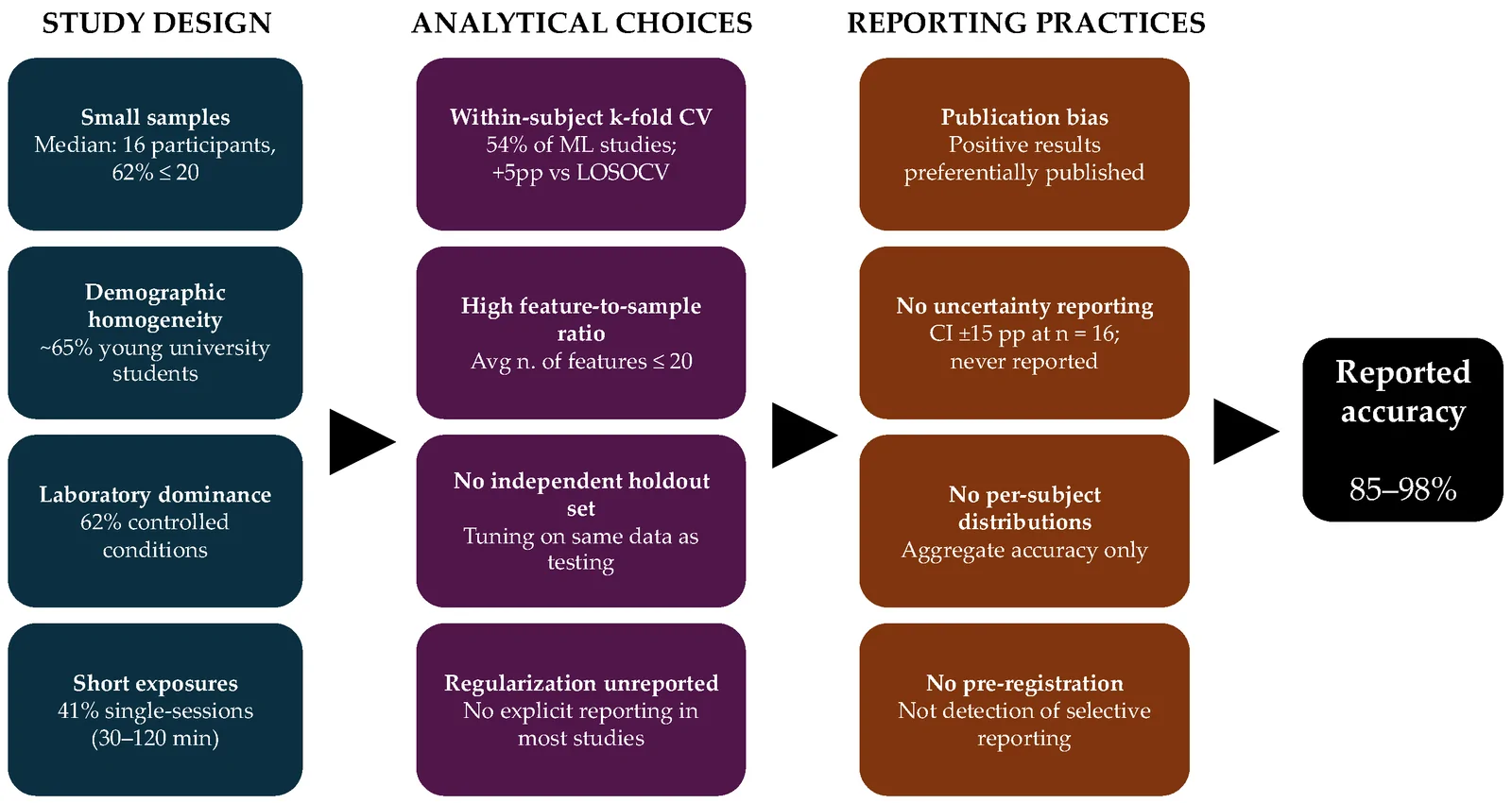
Sources of bias: study design constraints, analytical choices, and reporting filters, which compound progressively to inflate reported model performance.

The previous sections have mapped the current landscape of wearable sensing for thermal comfort across sensing technologies (Section 4), modeling approaches (Section 5), and building system integration (Section 6). This section synthesizes the gaps identified throughout the review, both those acknowledged by the authors of the reviewed studies and those that emerge from the cross-study analysis, and proposes prioritized research directions. The corpus exhibits several intersecting sources of bias that affect the reliability and generalizability of its findings. As illustrated in Figure 12, these biases operate at three levels and compound progressively to inflate reported performance relative to expected deployment accuracy.

### 7.1. Methodological Gaps

Small sample size is the single most frequently self-reported limitation in the corpus and the most consequential source of bias. The median sample is 16 participants, with 62% of studies enrolling 20 or fewer, a level insufficient for robust machine learning model training and undermining statistical power for detecting physiology–comfort associations in multivariate analyses. Small samples increase the variance of reported performance metrics, amplify the risk of overfitting (particularly for high-dimensional deep learning models), and produce unstable feature importance rankings. A simple binomial confidence interval on a reported accuracy of 90% with an effective sample size of 16 independent participants yields ±15 percentage points [173,174], a range never reported in the corpus.

More problematic than sample size alone is the demographic homogeneity of the participant pools. Approximately 65% of studies recruit from young university populations (20–30 years old), a demographic that is not representative of the broader building occupant population. This skew introduces systematic bias: younger adults have faster vasomotor responses, lower body fat percentages, and different clothing preferences than older populations. Only a handful of studies include vulnerable groups (children or elderly participants), and none specifically targets pregnant women, individuals with chronic conditions affecting thermoregulation (e.g., diabetes, cardiovascular disease, multiple sclerosis), or outdoor workers with prolonged heat exposure. Given that these populations face the highest thermal health risks, their near-total exclusion from the evidence base is a critical gap with implications for both scientific validity and health equity.

The tension between experimental control and ecological validity remains a persistent problem. Laboratory studies (62% of the corpus; 73 studies) provide precise control over thermal stimulus but impose artificial constraints (e.g., fixed clothing, restricted movement, short exposures) that limit transferability to real-world conditions. The 37 field studies (32%) provide higher ecological validity but also introduce confounding variables (activity, clothing changes, and environmental variability) and data quality challenges (motion artifacts, sensor dislodgment, missing data). The seven semi-controlled studies (6%) attempt a middle ground. The dominance of short-duration experiments further constrains the literature: thermal adaptation, circadian rhythms, and seasonal acclimatization operate on timescales of days to months that single-session studies cannot capture. Only a small number of studies exceed one month in duration, and even fewer combine longitudinal physiological monitoring with repeated subjective assessments, which is the combination needed to study how the physiology–comfort relationship evolves within individuals.

Beyond study design, several analytical choices further inflate reported performance. Validation strategy is the most consequential: as detailed in Section 5 and Table 4, validation strategy choice is associated with an indicative difference of approximately 5 percentage points in reported accuracy (k-fold CV: 90% median, IQR 79–94% vs. LOSOCV: 85% median, IQR 78–88%), yet 54% of ML studies use k-fold CV without acknowledging that this approach may inflate performance estimates for generalization use cases by allowing person-specific patterns to leak between training and test sets. Only eight studies (15% of the 52 ML studies [61,62,63,64,67,73,135,141]) employ LOSOCV, and even fewer report per-subject performance distributions (rather than just aggregate accuracy), which would reveal the inter-individual variability that aggregate metrics obscure.

Additional structural factors promote overfitting: high feature-to-sample ratios (many studies extract dozens of time-domain and frequency-domain features from very small samples), no explicit reporting of regularization, and the absence of holdout test sets independent of hyperparameter tuning in most k-fold studies. The field would benefit from adopting minimum reporting standards analogous to the TRIPOD guidelines [175] for prediction models in medicine, requiring LOSOCV validation (or equivalent inter-individual test), comparison against PMV/adaptive baselines, per-subject performance distribution, and explicit reporting of the number of output classes, dataset balance, and feature-to-sample ratio. On this last point, the comparison against established baselines is more encouraging: 11 studies (21% of ML studies [7,61,86,92,95,100,128,129,141,151,157]) compare their models against PMV or adaptive comfort predictions, though this still leaves most ML studies without a conventional benchmark.

At the reporting level, substantial heterogeneity across datasets, experimental conditions, and target variables introduces a further risk of comparability bias. Studies differ in environmental context (field or laboratory), comfort variable (thermal sensation, preference, or binary acceptability), number of output classes, and class distribution. Performance metrics such as accuracy are not directly equivalent across these problem formulations, yet they are routinely compared at face value. This heterogeneity is compounded by publication bias: positive results (high accuracy and improved comfort) are systematically more likely to be submitted and accepted than null or negative findings. The concentration of reported accuracies in the 85–98% range, with very few studies reporting below 70%, is consistent with publication bias [176], compounded by the methodological inflation associated with small-sample cross-validation [173,174]. However, it cannot be distinguished from genuine methodological improvement without access to unpublished data. The absence of registered study protocols across the entire corpus means pre-registration cannot be used to identify selective reporting.

Data availability emerges as a significant reproducibility barrier. Of 117 studies, only 9 make their datasets or code openly available, notably Gao et al. [132] (heterogeneous sensor dataset), Landa et al. [155] (IoT smartwatch open-technology platform), Mosteiro-Romero et al. [142] (smartwatch and urban datasets), Miller et al. [41] (JITAI open-source Cozie platform with GitHub repository), and Munir et al. [177] (depth sensor body shape dataset). This limited openness prevents independent validation, replication, and meta-analysis, and effectively means that most performance claims in the corpus cannot be independently verified. The contrast with adjacent fields is stark: in activity recognition and affective computing, public benchmark datasets (e.g., WESAD [178], PAMAP2 [179]) have accelerated methodological progress by enabling direct algorithm comparison. The thermal comfort community lacks an equivalent shared resource, though the open datasets from the studies cited above could serve as a foundation.

Overall, these intersecting risks (Figure 12) call for treating reported accuracy figures as upper-bound estimates of expected deployment performance rather than as stable population parameters.

The methodological risks described above can be quantified: Table 8 reports the ten quality indicators defined in Section 2 across the whole corpus. First, only nine studies (8%) make data or code openly available; a further 34 state that data are available from the authors on request. Second, 53 studies (45%) report ethical approval and a further 17 report informed consent without reference to an approving body, leaving 47 studies (40%) that report neither. Third, only 2 studies (2%) report a quantitative figure for the proportion of data excluded or retained after preprocessing, and only 41 (35%) describe their preprocessing at all. A controlled head-to-head comparison, in which sensing configurations and model families are evaluated on a common dataset under a fixed task, would settle several of the questions raised in Section 5 directly. It cannot, however, be assembled from the published record: only 9 of the 117 studies release their data, and those that do differ in the signals acquired, the sampling rates, the comfort scales and the populations studied, so that no common evaluation set exists. The comparisons reported in Section 5 are accordingly stratified rather than controlled, and open benchmark datasets are the first item of the research agenda in Section 7.5.

### 7.2. Technological Gaps

The device landscape described in Section 4 reveals a heterogeneous mix of research-grade, consumer, and custom devices with widely varying signal quality. Cross-study comparison is undermined by the absence of inter-device calibration: an EDA value from the Empatica E4 (ventral wrist, stainless steel electrodes, 4 Hz) is not directly comparable to one from a custom finger-mounted sensor (palmar surface, Ag/AgCl electrodes, 128 Hz), nor to an ErgoLAB wireless sensor (used in [43]) or a BIOPAC MP160 system (used in [98]). Standardized calibration protocols, comparable to the ISO 7726 framework for environmental sensors, do not exist for physiological wearables in the comfort domain.

Moreover, the reviewed studies reveal an inverse relationship between sensing richness and deployment duration. The most comprehensive multi-signal configurations (EEG + ECG + EDA + SKT, using 3–4 separate devices, e.g., [86,91,92]) are limited to sessions of a few hours, while the longest field deployments (weeks to months) use minimal sensing (a single iButton or smartwatch). This trade-off between signal diversity and wearability duration remains unresolved, and emerging integrated platforms (e.g., Empatica EmbracePlus used in [19], next-generation smartwatches with EDA) may partially address it, but only if their measurement quality approaches research-grade standards.

Only 29% of the corpus (34 studies) uses dedicated near-body environmental sensors, despite the demonstrated benefit (Section 5) that combining physiological and environmental features improves prediction accuracy by approximately 4 percentage points over physiological data alone. The integration of compact, low-power environmental sensors (temperature, humidity, and air velocity) into wearable platforms, or into complementary body-worn devices such as badges or clothing clips, could substantially improve prediction performance with minimal additional participant burden. The spatial mismatch between the location of environmental measurements and the placement of physiological sensors (Section 4.5) also warrants systematic investigation. A further source of uncertainty that receives almost no attention is the quality of sensor measurements. Skin temperature readings from infrared and thermistor-based wearables are sensitive to ambient air currents, contact pressure, and peripheral vasoconstriction; heart rate derived from PPG is vulnerable to motion artifacts, particularly during ambulatory measurements; electrodermal activity signals require careful electrode placement and skin preparation protocols that are rarely standardized across studies. Calibration drift over multi-day deployments is reported anecdotally but not quantified systematically in any of the reviewed studies. These sources of physiological variability and instrument noise are not independent of study design choices: short laboratory sessions minimize drift and motion artifacts, whereas field studies must contend with them simultaneously. Future work should report signal quality metrics (e.g., the percentage of data rejected due to motion artifacts and inter-device variability in concurrent measurements) as part of standard data documentation. Viewing the corpus through a Technology Readiness Level (TRL) lens clarifies the maturity distribution of the evidence base. The three studies that implement closed-loop wearable-informed HVAC control [24,65,68] represent a TRL 4–5 (technology validated in a laboratory or relevant environment). The roughly 80 studies that discuss energy implications conceptually without implementation or simulation sit at TRL 1–3 (basic principles observed; technology concept formulated). No study in the corpus reaches TRL 6 or above (prototype demonstration in an operational building environment at a realistic scale). This framing has direct implications for how the energy findings should be read: the evidence base supports the technical feasibility of wearable-informed building control at the laboratory scale. However, it does not yet support quantified projections of real-world energy savings.

### 7.3. Analytical Gaps

With only three studies addressing transfer learning or domain adaptation [133,141,167], this represents the most critical analytical gap for practical deployment. Any wearable comfort system installed in a real building must handle new occupants without requiring days of labeled calibration data, adapt to seasonal climate shifts without retraining, and generalize across building types with different HVAC configurations. The transfer learning studies in the corpus demonstrate technical feasibility but are too few, too small, and too heterogeneous to support deployment guidelines. This gap contrasts with adjacent fields (activity recognition, emotion detection), where cross-person and cross-context transfer is an active and mature research area with established benchmarks and methodological frameworks.

The ML studies in the corpus predominantly treat comfort prediction as a black-box classification problem, reporting aggregate accuracy without analyzing which features drive predictions and under what conditions. This approach limits both scientific insight (understanding the physiological mechanisms of thermal comfort) and practical utility (knowing which sensors are essential versus redundant for a given deployment context). Feature importance analysis (e.g., using SHAP values, permutation importance, or partial dependence plots) would enable rational sensor selection, reduce deployment costs and participant burden, and maintain prediction quality. Only a handful of studies in the corpus conduct systematic feature importance analysis (e.g., [46,61,80,91]), and the use of modern explainability methods remains rare.

Moreover, most ML studies in the corpus treat each data point as independent, extract features from fixed-duration windows, and classify them in isolation. This approach ignores the temporal dynamics of comfort transitions: the physiological process by which a person shifts from comfortable to uncomfortable (or vice versa) happens over minutes, with characteristic time constants that differ across signals (SKT responds in seconds, HRV in minutes, EDA in tens of seconds). Temporal models (e.g., LSTM, GRU, temporal CNNs, or attention-based architectures) could capture these transition dynamics, enabling predictive (rather than reactive) comfort control. The eight deep learning studies in the corpus [62,74,100,130,133,140,141,166] represent a start, but none explicitly model comfort transitions as a sequential prediction problem. Equally underexplored are the choices of temporal window length and lag structure used in feature extraction. Across the corpus, window lengths range from a few seconds to several minutes, and most studies select these values empirically without justification or sensitivity analysis. The optimal window depends on the signal: short windows (5–10 s) capture transient EDA responses, while longer windows (1–5 min) are needed to stabilize HRV frequency-domain features. Lag effects (i.e., the delay between a thermal stimulus and the physiological response it stimulates) are similarly unaddressed: studies that assign a comfort label to a contemporaneous physiological window may be misaligned with the actual response time of the signal. Standardizing window and lag conventions, or treating them as explicit hyperparameters reported alongside model accuracy, would substantially improve cross-study comparability and could close some of the unexplained variance in reported performance.

### 7.4. Application Gaps

As detailed in Section 6, the pathway from comfort prediction to building system control remains the weakest link in the research pipeline. The field needs standardized wearable-to-BMS communication protocols, multi-occupant aggregation algorithms tested in realistic shared-zone configurations, degraded-mode operation strategies for partial wearable adoption, and quantified energy impact assessments using building simulation tools. Moreover, continuous physiological monitoring in a workplace produces data that are health data in substance: heart rate variability, electrodermal activity, and skin temperature are the signals on which the literature on stress, cognitive load and affect is built, so a dataset assembled to infer thermal sensation also supports inferences about arousal, fatigue and emotional state that formed no part of the purpose for which participation was agreed. Under the European General Data Protection Regulation, such data fall within the special categories of Article 9, and in an occupational setting the difficulty is not the legal basis in the abstract but the validity of consent, since the asymmetry between employer and employee makes freely given consent difficult to establish. The design question is therefore how to obtain the control benefit without acquiring the data, and three strategies may answer it, namely on-device inference, feature minimization, and federated learning. Privacy is in this sense a determinant of feasibility and not only an ethical constraint: no reviewed study measures occupant willingness to be monitored, although it plausibly limits deployment duration as much as battery life does.

The 9 outdoor-only studies [17,43,97,101,125,145,165,171,172] represent a nascent but important direction. Wearable sensing offers unique capabilities for pedestrian thermal exposure assessment: continuous physiological monitoring during movement through varied microclimates, validation of urban simulation models at the human scale (e.g., Ignatius et al. [145]), and personalized heat-stress warning systems for outdoor workers. The outdoor domain also presents unique sensing challenges: solar radiation exposure, wind variability, and high activity levels generate signal artifacts that indoor studies do not encounter. The connection between outdoor exposure and indoor comfort (the thermal history effect, Section 6) is a particularly promising but unexplored integration opportunity.

In terms of standardization, at least 20 distinct comfort scale formulations appear in the corpus, with TSV (thermal sensation vote, 78 occurrences), TP/TPV (thermal preference vote, 30 occurrences), TC (thermal comfort, 27 occurrences), and TCV (thermal comfort vote, 14 occurrences) as the most common. Scale granularity ranges from binary (comfortable/uncomfortable) to continuous, and the relationships among sensation (how the environment feels), preference (what change is desired), and comfort (overall satisfaction) are operationalized differently across studies. This heterogeneity prevents meaningful cross-study performance comparison and complicates meta-analysis. A community-wide consensus on a minimum set of standardized comfort scales, analogous to the ASHRAE 7-point sensation scale but extended to preference and acceptability, would substantially improve the comparability and cumulative value of future research.

### 7.5. Future Steps

Based on the gap analysis above, the following priorities are proposed:

- The creation and public release of large-scale, multi-site, demographically diverse open benchmark datasets on wearable comfort, with standardized physiological signals, environmental measurements, and comfort labels, would enable direct algorithm comparison, reproducibility verification, and methodological progress. The existing open datasets from [41,132,142,155,177] provide a foundation but are insufficient in scale and diversity.
- Standardizing validation and reporting (e.g., adopting LOSOCV validation as a minimum standard, routine comparison against PMV/adaptive baselines, building on the 11 studies that already do so, with the adaptive model rather than PMV used as the reference for studies conducted in naturally ventilated or mixed-mode buildings, and per-subject performance reporting) would make the literature interpretable and comparable. As Table 4 demonstrates, the validation strategy is associated with an indicative 5-percentage-point difference in reported accuracy. However, this comparison should be interpreted cautiously, given the heterogeneity of tasks, datasets, and class structures across studies.
- Studies should report the proportion of data retained after preprocessing, the criteria by which windows were excluded, and, for optically and electrically acquired signals, motion-artifact rejection rates. A reported accuracy computed on an unstated fraction of the collected data is not interpretable, and a model that appears robust may simply have been evaluated on the cleanest segments of a recording. This is the least expensive of the changes proposed here, since the information already exists within the analysis pipelines of the studies concerned and only needs to be reported. As Table 8 records, only 2 of the 117 studies reviewed here currently do so.
- Systematic investigation of cross-person, cross-building, and cross-climate model transfer, including few-shot personalization methods that calibrate a general model to a new individual with minimal labeled data, would bridge the gap between laboratory demonstrations and practical deployment. The three existing transfer learning studies [133,141,167] demonstrate feasibility but represent a critically thin evidence base.
- Foundation models for physiological time series, pre-trained on large unlabeled corpora of wearable recordings and fine-tuned on small comfort-labeled datasets, offer a structural response to the constraint that limits almost every study in the corpus, namely a median cohort of 15 participants. Suitable pre-training corpora already exist in adjacent domains such as activity recognition, sleep staging and affective computing, and the transfer studies in the corpus establish that cross-domain pre-training is feasible in principle [180]. Whether representations learned from such corpora carry thermally relevant information is a well-defined question that the field has not yet asked. Multimodal architectures that jointly encode physiological, near-body environmental and contextual streams are the natural counterpart on the fusion side and would replace the hand-designed feature concatenation that characterizes the corpus.
- Federated learning would allow models to be trained across occupants, buildings and climates without centralizing physiological records, addressing simultaneously the transfer problem identified above and the governance problem set out in Section 7.4. Differential privacy and secure aggregation are the corresponding techniques for bounding what can be reconstructed from model updates [181]. No study in the present corpus adopts either, although both are established in adjacent health-sensing studies.
- Edge inference, executed on the wearable or on a local gateway rather than in the cloud, is the enabling condition for the privacy strategy above and for the long-duration field campaigns the field lacks. It imposes a power and memory budget that favors the simpler models discussed in Section 5.6, and quantifying the accuracy cost of operating within that budget is a practical research problem that the corpus does not address.
- Moving beyond single-occupant proof-of-concept studies to multi-occupant, multi-zone implementations that address aggregation, equity, and partial-adoption scenarios would demonstrate practical viability for building operators. Only one study (Deng & Chen [65]) has addressed multi-occupant HVAC optimization; this must become the norm rather than the exception. Multi-occupant aggregation requires concrete algorithmic proposals: rule-based aggregation over the predicted comfort distribution, for instance satisfying a minimum acceptable proportion of occupants; multi-objective optimization under explicit fairness constraints, minimizing maximum individual discomfort rather than mean discomfort; social-choice and voting formulations applied to predicted preference distributions, which carry a well-developed theory of the trade-offs they entail; multi-agent reinforcement learning with a reward combining aggregate comfort and energy use; and dynamic zoning, in which occupants are grouped by predicted preference and zone setpoints follow the resulting clusters. Each option embeds a different normative assumption about what a fair distribution of thermal comfort is, and a controller minimizing mean discomfort and one minimizing maximum discomfort will systematically favor different occupants. The corpus treats aggregation as a purely technical problem, but it should instead be reported and justified as a design decision with distributive consequences.
- Digital twin integration, present in only two studies of the corpus, would allow wearable-derived comfort data to drive what-if analysis within a calibrated building or urban model, making it possible to estimate the energy and comfort consequences of a control strategy before committing to it. This relates directly to the scarcity of quantified energy outcomes identified in Section 6, which is the gap that most limits the practical uptake of this corpus.
- Occupant-derived comfort state has no standard representation in the common building data models: neither BACnet object types nor semantic schemas, such as Brick Schema [182] and Project Haystack [183], define an occupant comfort point carrying an associated confidence, provenance, and validity interval. Until such a representation exists, every wearable-to-BMS integration must be built bespoke, which is a sufficient explanation for why only three studies in the corpus have completed one. Proposing and standardizing such a point type is a critical intervention for the feasibility of closed-loop studies.
- Targeted studies involving elderly adults, children, outdoor workers, and individuals with thermoregulatory impairments would extend the evidence base to the populations most vulnerable to thermal extremes and most in need of personalized comfort interventions.

## 8. Conclusions

This systematic review examined 117 studies employing wearable sensing for thermal comfort assessment in and around buildings, following a PRISMA 2020-compliant protocol with AI-assisted screening and structured data extraction across 24 variables per study.

This review advances the current state of knowledge by bridging three dimensions that have typically been treated separately in the literature: wearable sensing technologies, personal comfort modeling, and building system integration. By combining a comprehensive hardware analysis, a structured cross-study evaluation of modeling approaches, and a critical assessment of validation practices and energy implications, this work provides a unified perspective on wearable-based thermal comfort research. In doing so, it not only consolidates existing knowledge but also highlights key methodological and practical barriers that must be addressed to enable real-world deployment.

The corpus reveals a field in rapid growth with a maturing technological foundation and increasingly sophisticated analytical methods. Skin temperature and heart rate dominate the physiological sensing landscape (77 and 69 studies, 66% and 59% respectively), measured predominantly at the wrist (70 studies, 60%) using commercial platforms such as the Empatica E4 (18 studies), iButton family (21 studies), and consumer smartwatches (24 studies). Machine learning models, employed by 52 studies (44%), achieve a median classification accuracy of 90%, with ensemble methods (Random Forest, gradient boosting) and SVM providing the most reliable performance across all output categories. Combined physiological and environmental features are associated with higher reported accuracy inputs by approximately 4 percentage points, supporting the multimodal sensing paradigm adopted by 71% of the corpus.

However, the review also exposes significant structural limitations. Most studies rely on small, demographically homogeneous samples (median: 16 participants), drawn predominantly from young university populations. The predominance of short-duration laboratory experiments (62% of the corpus) limits ecological validity, and only 37 field studies (32%) capture comfort under naturalistic conditions. Validation practices are inconsistent: k-fold cross-validation is associated with approximately 5 percentage points higher reported accuracy compared to the more conservative leave-one-subject-out approach (90% vs. 85% median, IQR: 79–94% vs. 78–88%), a difference that likely reflects the contribution of individual-specific patterns rather than true generalization, yet 54% of ML studies rely on k-fold alone. Twenty-one percent of ML studies (11 of 52) compare their models against PMV baselines, an encouraging but still insufficient proportion for establishing the practical value proposition of wearable sensing against the established standard.

The most critical gap lies in the pathway from prediction to practice. While most studies discuss HVAC or energy implications at a conceptual level, only five reach the building-actuation stage, of which three implement closed-loop wearable-informed control and only one reports a specific quantified energy outcome (76% reduction with localized heating). The multi-occupant aggregation problem, fundamental to any real-world deployment in shared spaces, is addressed by a single study. Only three studies explore transfer learning across individuals and contexts, which is essential for scalable deployment. Open data sharing remains limited, though a small number of studies have made datasets or code publicly available, providing a nascent foundation for reproducibility.

These findings lead to five actionable recommendations for the research community and practitioners:

- The creation and release of open benchmark datasets with standardized signals, scales, and demographic diversity would accelerate methodological progress more than any algorithmic innovation.
- The adoption of LOSOCV validation and PMV benchmarking as minimum reporting standards, together with benchmarking against the adaptive comfort model for field studies and routine reporting of signal quality and data retention, would make the literature interpretable and actionable.
- Systematic investigation of transfer learning and few-shot personalization methods would bridge the gap between laboratory accuracy and real-world viability.
- Multi-occupant integration studies in realistic building configurations that address aggregation algorithms, equity considerations, and partial-adoption scenarios would demonstrate practical viability for building operators and facilities managers.
- Targeted inclusion of vulnerable populations would expand the evidence base for those who need personalized thermal management most and face the highest thermal health risks.

The trajectory from wearable data to adaptive, energy-efficient building operation is technically feasible, as demonstrated by the five implemented studies. Realizing this trajectory at scale requires a shift in research emphasis: from optimizing individual algorithms to building reproducible, generalizable, and deployable comfort sensing systems that serve diverse occupants in real buildings.

## Figures and Tables

**Table 1:** Positioning of the present review with respect to the most closely related previous reviews on wearable and personal thermal comfort sensing.

Review (Year)	Design	Coverage	Sensing Hardware	Comfort Modeling	Validation Analysis	Building and Energy Integration	Outdoor/Urban
Čulić et al. [25] (2021)	Narrative	2017–2021	Broad taxonomy of monitoring technologies (environmental, camera-based, wearable)	Not analyzed	Not addressed	Conceptual	Not covered
Mansi et al. [16] (2021)	Narrative	to 2021	Physiological signal acquisition and sensor reliability	Signal processing only	Not addressed	Not addressed	Not covered
Feng et al. [23] (2022)	Systematic	previous 10 years (to 2021)	Partial	Data-driven prediction pipeline	Partial	Conceptual	Not covered
Arakawa Martins et al. [14] (2022)	Systematic	to 2021	Partial	Personal comfort models, all input types	Partial	Partial	Not covered
Costantino et al. [27] (2023)	Systematic	2012–2022	Off-the-shelf devices, detailed	Not analyzed	Not addressed	Not addressed	Not covered
Gao and Ghahramani [20] (2026)	Systematic	to 2024	Sensing systems and data preprocessing	Modeling approaches	Partial	Not addressed	Not covered
This review (2026)	Systematic	2008–2026 (no temporal restriction)	Device families mapped to modeling constraints	52 ML studies stratified by output category	Dedicated cross-study analysis	80 studies; 5 reaching actuation	9 outdoor studies

**Table 3:** Summary of the 52 ML-based personal comfort modeling studies by output category.

Output Category	Number of Studies	Number of Subjects	Dominant Physiological Signals (n)	Most Common ML Methods (n)	Validation Methods (n)	Accuracy/F1	R^2^ or MAE	PMV Comp.	Output Category
TSV/ thermal sensation (3-class)	8 [24,64,74,86,90,92,126,133]	range: 3–51; median: 11	SKT (8); HR (5); HRV (3); EDA (2); BVP (2); ECG (2)	SVM (8); RF (4); KNN (3); GBM (2); DL (2)	5-fold CV (3); hold-out (3); LOSOCV (1); 10-fold CV (1)	range: 83–96%; median: 90%	Not reported	[86,92]	TSV/thermal sensation (3-class)
TSV/ Comfort (multi-class: 5–10 pt)	10 [42,67,84,98,128,136,150,151,159,166]	range: 10–52; median: 15	SKT (9); HR (7); EDA (4); EEG (1)	RF (6); ANN (6); SVM (6); KNN (4); DT (3)	10-fold CV (4); hold-out (3); 5-fold CV (2); LOSOCV (1)	range: 77–98%; median: 90%	MAE = 0.47 [151]	[128,151]	TSV/comfort (multi-class: 5–10 pt)
Comfort/ discomfort (binary)	10 [62,79,80,91,99,100,111,134,153,154]	range: 5–76; median: 20	SKT (8); EDA (5); EEG (4); HRV (4); PPG (3)	SVM (8); RF (6); ANN (3); NB (3); KNN (3)	10-fold CV (5); 5-fold CV (3); LOSOCV (1); hold-out (1)	range: 67–94%; median: 86%	MAE = 3.12 [99]	[100]	Comfort/discomfort (binary)
Thermal preference (3-class)	8 [7,18,44,55,88,123,125,135]	range: 2–30; median: 17	SKT (8); HR (8); EDA (4); ACC (2); HRV (1)	SVM (6); GBM (6); RF (5); KNN (5); ANN (3)	5-fold CV (6); LOSOCV (2)	range: 71–99%; median: 79%	Not reported	[7,61,129,141]	Thermal preference (3-class)
TSV (regression)	5 [63,65,95,158,167]	range: 5–24; median: 15	SKT (5); HR (3); HRV (2); EDA (1)	LinReg (3); RF (1); DT (1); SVM (1); ET (1)	Hold-out (3); LOSOCV (1); 5-fold CV (1)	Not reported	R^2^: 0.72–0.99; MAE: 0.86 [63]; RMSE: 0.82 [167]	[95]	TSV (regression)
PMV index estimation	2 [156,157]	range: 4; median: 4	SKT (2); HR (2); EDA (1)	ANN (2)	Hold-out (2)	Not reported	R^2^: 0.999; RMSE: 0.023	[157]	PMV index estimation
PMV input estimation (MET, clo)	3 [40,140,152]	range: 1–23; median: 20	HR (2); SKT (1); heat flux (1)	DL (1); DT (1); LinReg (2)	Hold-out (3)	range: 88–95%; median: 89%	Not reported	Not reported	PMV input estimation (MET, clo)
Other (core temp., heat event, sleep comfort, JITAI)	6 [41,73,93,121,130,135]	range: 7–103; median: 19	SKT (3); HR (3); HRV (3); EDA (2)	RF (4); SVM (2); Clustering (1); Kalman (1)	Mixed	range: 85–99%	R^2^: 0.74–0.85 [73,135]	Not reported	Other (core temp., heat event, sleep comfort, JITAI)

Note: Signals show frequency of use across studies in the category. PMV comparison = studies that explicitly compared ML model performance against PMV. Accuracy reported as classification accuracy or F1 score (whichever was the primary metric).

**Table 4:** Impact of validation strategy on reported model accuracy (classification studies only).

Validation Strategy	Number of Studies	Accuracy Range	Median Accuracy	Mean Accuracy	Number of Subjects (Median)	Key Observation	Validation Strategy
LOSOCV (leave-one-subject-out)	6 [61,62,64,67,135,141]	71–92%	85%	83%	16	Tests true generalization to unseen individuals; consistently lower accuracy; most conservative and realistic estimate of deployment performance	LOSOCV (leave-one-subject-out)
Within-subject k-fold CV (5-fold or 10-fold)	25 [7,19,24,42,50,74,79,80,90,91,92,94,98,111,121,128,129,130,131,134,136,150,151,153,154]	67–99%	90%	88%	14	Trains and tests on the same subjects; higher reported accuracy due to learned individual patterns; risk of overfitting to participant-specific behavior	Within-subject k-fold CV (5-fold or 10-fold)
Hold-out split (train/test partition)	8 [40,84,86,100,126,133,159,166]	73–99%	90% (IQR: 86–96%)	90%	16	Single random split; no cross-validation robustness; results sensitive to partition; highest reported accuracies but least reproducible	Hold-out split (train/test partition)
LOSOCV + regression (MAE/RMSE reported)	2 [63,73]	—	—	—	46	MAE: 0.86 TSV points [63]; MAE: 0.1 °C core temp/0.3 TSV [73]; regression metrics more interpretable than classification accuracy	LOSOCV + regression (MAE/RMSE reported)
Δ LOSOCV vs. within-subjectCV			Δ = −5 pp	Δ = −5 pp	—	The 5-percentage-point gap between LOSOCV (85% median) and within-subject CV (90% median) represents the accuracy gained by learning individual patterns that does not transfer to new users	Δ LOSOCV vs. within-subjectCV

Note: Only studies reporting classification accuracy or F1 score are included.

**Table 5:** Validation strategy selection framework for personal thermal comfort models.

Modeling Objective	Intended Deployment	Recommended Validation	Rationale
Generalization (the model must work for new users with no prior data)	Building-wide deployment. New occupant on the first day	LOSOCV	Simulates true inter-individual prediction; no identity leakage between train and test
Personalization (the model is calibrated on each user’s own historical data)	Adaptive system with an onboarding phase	Within-subject k-fold CV	User identity is legitimately shared; tests intra-individual consistency
Domain adaptation (the model is pre-trained and fine-tuned on a new user)	Transfer learning/few-shot calibration	LOSOCV for pre-training + held-out user data for fine-tuning	Evaluates both the population model and the adaptation gain separately
Regression/continuous output	Any of the above	MAE/RMSE with LOSOCV; report per-subject distribution	Accuracy is not meaningful for regression; LOSOCV still applies

**Table 6:** Reported classification performance stratified by task formulation and sensing configuration.

Task Formulation	Sensing Configuration	Studies (n)	Accuracy Range	Median Accuracy	Median Sample Size	Validation Protocols (n)
TSV/thermal sensation (3-class)	Physiological only	1	94%	94%	8	k-fold CV (1)
TSV/thermal sensation (3-class)	Physiological + environmental	7	83–96%	87%	11	k-fold CV (3); hold-out (2); LOSOCV (1); other (1)
TSV/comfort (multi-class, 5–10 pt)	Physiological only	4	77–97%	88%	32	hold-out (2); k-fold CV (1); LOSOCV (1)
TSV/comfort (multi-class, 5–10 pt)	Physiological + environmental	6	79–98%	91%	14	k-fold CV (5); hold-out (1)
Comfort/discomfort (binary)	Physiological only	4	74–93%	81%	17	k-fold CV (3); hold-out (1)
Comfort/discomfort (binary)	Physiological + environmental	6	67–94%	90%	18	k-fold CV (5); LOSOCV (1)
Thermal preference (3-class)	Physiological only	3	73–96%	80%	18	k-fold CV (2); LOSOCV (1)
Thermal preference (3-class)	Physiological + environmental	5	71–99%	78%	16	k-fold CV (4); LOSOCV (1)

Note: “physiological only” indicates that no environmental variable enters the feature set.

**Table 7:** The five studies reaching the building-actuation stage, classified by the depth of implementation achieved.

Study	Depth of Implementation	Setting and Occupancy	Subjects (n)	Actuation	Quantified Energy Outcome
Yoshikawa et al. [67]	Sensing and prediction within a control use case	Laboratory, office use case	15	Estimation evaluated against an air-conditioning use case; the control action is not exercised in the reported experiment	Not reported
Hu et al. [66]	System-level platform, occupant-directed actuation	Semi-controlled shared office	30	Cloud data fusion, edge processing and BMS-level architecture; the reinforcement-learning layer is directed at occupant behavior rather than at the building system	Not reported
Ren et al. [68]	Closed loop on a personal comfort system	Laboratory chamber, single occupant	16	Activity-dependent localized heating driven by physiological feedback	In total, 75.6% heating energy reduction against conventional HVAC
Cho et al. [24]	Closed loop on HVAC, proof of concept	Laboratory chamber, single occupant	7	Wearable prediction directly adjusts the room temperature setpoint	Reported qualitatively (reduced consumption); no figure given
Deng and Chen [65]	Closed loop on HVAC, field validation	Field, multi-occupant offices	24	Individual predictions aggregated into a shared zone setpoint	Not reported; comfort outcome only

**Table 8:** Quality indicators across the 117 studies of the corpus.

Indicator	Condition Recorded	No. of Associated Studies
Sample size	At least 30 participants	29/117 (25%)
Setting	Field or semi-controlled rather than laboratory	44/117 (38%)
Near-body sensing	Dedicated near-body environmental sensors used	34/117 (29%)
Sampling rate	Sampling rate reported for the acquired signals	116/117 (99%)
Validation	Cross-subject validation, among the 52 ML studies	8/52 (15%)
Conventional baseline	Model compared against PMV or the adaptive model	11/117 (9%)
Demographics	Participant age and sex both reported	72/117 (62%)
Open data	Data or code openly available (no “on request”)	9/117 (8%)
Data retention	Quantitative figure for data excluded or retained after preprocessing	2/117 (2%)
Ethical approval	Ethical approval reported	53/117 (45%)

## Data Availability

The full database, including all 24 extraction variables and the 10 quality indicators across 117 studies and the ASReview project file documenting the complete screening history, are provided as Supplementary Material.

## Supplementary Materials

The following supporting information can be downloaded at https://www.mdpi.com/article/10.3390/s26175640/s1. Table S1: Full database, including all 24 extraction variables and 10 quality binary indicators across 117 studies; File S1: ASReview project file containing the complete screening history, the model configuration of each round, and the final ranking.

## Abbreviations

The following abbreviations are used in this manuscript:

ACC	Accelerometry
ANN	Artificial neural network
AUC	Area under the curve
BACnet	Building automation and control networks
BG	Blood glucose
BLE	Bluetooth low energy
BMS	Building management system
BP	Blood pressure
BPNN	Back-propagation neural network
BVP	Blood volume pulse
CatBoost	Categorical boosting
CNN	Convolutional neural network
CO_2_	Carbon dioxide
CV	Cross-validation
DL	Deep learning
DNN	Deep neural network
DT	Decision tree
ECG	Electrocardiography
EDA	Electrodermal activity
EDL	Electrodermal level
EDR	Electrodermal response
EEG	Electroencephalography
EMG	Electromyography
ET	Extra trees
F1	Harmonic mean of precision and recall
fEMG	Facial electromyography
GBM	Gradient boosting machine
GRU	Gated recurrent unit
GSR	Galvanic skin response
HF	High-frequency power
HR	Heart rate
HRV	Heart rate variability
HVAC	Heating, ventilation, and air conditioning
IAQ	Indoor air quality
IBI	Inter-beat interval
ICA	Independent component analysis
IoT	Internet of Things
IR	Infrared
JITAI	Just-in-time adaptive intervention
KNN	K-nearest neighbors
LF	Low-frequency power
LightGBM	Light gradient boosting machine
LOSO	Leave-one-subject-out
LR	Logistic regression
LSTM	Long short-term memory
MAE	Mean absolute error
MET	Metabolic equivalent of task
ML	Machine learning
MLP	Multi-layer perceptron
MRT	Mean radiant temperature
NB	Naive Bayes
NTC	Negative temperature coefficient
OTS	Overall thermal sensation
PCS	Personal comfort system
PET	Physiological equivalent temperature
PM2.5	Particulate matter ≤2.5 μm
PM10	Particulate matter ≤10 μm
PMV	Predicted mean vote
pNN50	Perc. of intervals differing by more than 50 ms
PPD	Predicted percentage dissatisfied
PPG	Photoplethysmography
PSD	Power spectral density
R^2^	Coefficient of determination
RF	Random forest
RH	Relative humidity
RMSE	Root mean square error
RMSSD	Root mean square of successive differences
RNN	Recurrent neural network
SC	Skin conductance
SCL	Skin conductance level
SCR	Skin conductance response
SDNN	Standard deviation of NN intervals
SHAP	SHapley Additive exPlanations
SKT	Skin temperature
SMAT	Selective multi-source adaptation for thermal comfort
SpO_2_	Blood oxygen saturation
SVM	Support vector machine
TC	Thermal comfort
TCV	Thermal comfort vote
TP	Thermal preference
TSV	Thermal sensation vote
TVOCs	Total volatile organic compounds
XGBoost	Extreme gradient boosting

## Appendix A

This appendix contains the main details of all 117 studies included in this review.

**Table A1:** Main details of the review’s corpus.

Ref	Country	Environment	Wearable Devices	Physiological Signals	Comfort Scale	Modeling Approach	Output Variable	Validation Method	Validation Results
[7]	Singapore	Indoor	Fitbit Versa; iButtons model DS1925	SKT; HR	TP	SVM; Random Forest; XGBoost; Multi-Layer Perceptron; Logistic Regression; K-Nearest Neighbors; Gaussian Naive Bayes	Thermal preference (3-class: warmer/no change/cooler)	Random search with 5-fold cross-validation, repeated 100 times for each model	Median F1 = 0.78; PMV Comparison
[17]	Italy	Outdoor	Custom 3D printed station on helmet/backpack	None	None	Qualitative cluster analysis	Intra-urban environmental variability	Comparison with reference microclimate station	Good linear correlation for lighting (R^2^ = 0.98), CO_2_ (R^2^ = 0.92), and air temperature/RH overlapping error bars
[19]	USA	Indoor	Empatica EmbracePlus	SKT; HR; EDA	TSV; TP; perceived heat intensity	Random Forest; SVM; KNN; ANN	Heat intensity (5-point scale); thermal sensation (10-point scale); thermal preference (7-point scale)	5-fold cross-validation with hyperparameter optimization	Accuracy: F1 = 0.96; AUC: 0.99
[24]	South Korea	Indoor	Custom soft wearable sensor	SKT	Thermal comfort questionnaire	Random Forest; Linear Regression; Logistic Regression; Support Vector Machine; Ridge Classifier; Logistic Discriminant Analysis; Support Vector Classifier; Gradient Boosting Machine; K-Nearest Neighbor	Thermal comfort (3-class: cool discomfort, comfort, warm discomfort)	5-fold CV	Accuracy: 93.9%
[40]	China	Indoor	Custom metabolic rate sensor; MAX30102 chip; InBody Dial H20N analyzer scale	HR; skin resistance; heat loss; body muscle rate	None (metabolic rate measurement study; no comfort survey)	Linear regression model	Metabolic rate (Met)	comparison against Quark CPET indirect calorimetry (gold standard)	95% accuracy; R^2^ > 0.91 across different temperature conditions
[41]	Singapore	Both	Apple Watch	HR; sound level; step count; walking distance; standing time; oxygen saturation	Thermal preference; noise distraction	Random Forest	Thermal preference (3-class: prefer warmer/no change/prefer cooler); noise preference (5-class)	3-fold cross-validation	3–13% increase in behavior adjustments for thermal comfort; 4–11% for noise location changes
[42]	USA	Indoor	SBS-BTA surface temperature sensors	SKT	TSV	J48 decision tree; stepwise regression	Overall Thermal Sensation (OTS) (5-point scale from −2 to 2)	10-fold CV	Accuracy: 97.44%; R^2^: 55.76%
[43]	China	Outdoor	ErgoLAB wireless sensors	SKT; HRV; SC	TSV; TCV; RSA; ASV; HSV; WSV	Wilcoxon signed-rank test for statistical significance and correlation analysis	Changes in physiological wellbeing and psychological comfort votes	Comparison between landscape microclimate (shaded) and open space (control)	Landscape microclimates significantly decreased skin temp (−0.3 °C) and improved HRV indices (LF/HF ratio)
[44]	China	Indoor	Powerlab 8/30 system (ADInstruments, Australia)	ECG; HRV	TSV; TC	HRV Spectral Analysis (LF/HF ratio)	Thermal comfort level	Two-way ANOVA and paired-samples *t*-test	LF/HF at discomfort level significantly higher than at comfort level (*p* < 0.05) even with same thermal sensation
[45]	South Korea	Indoor	Empatica E4	EDA; HR; SKT	TP	Multilevel modeling (MLM)	Thermal preference votes (want warmer; no change; want cooler)	Likelihood ratio test, Bootstrapping, *t*-tests on regression coefficients	EDL is a significant predictor of thermal preference (*p* < 0.001) while SKT is less significant after controlling other signals
[46]	Italy	Indoor	Interaxon Muse 2-Bundle; Empatica E4	EEG; HRV; EDA; SKT	TSV	Statistical analysis (ANOVA, Kruskal–Wallis, Dwass–Steel–Critchlow–Fligner)	Thermal sensation (3-class: cold/neutral/warm)	Cross-validation test using Mann–Whitney U non-parametric *t*-test	Significant differences (*p* < 0.01) between cold and warm for Beta TP10, Gamma TP10, LF/HF, EDA tonic, and mean ST
[47]	China	Both	iButton DS1922L; YUYUEYHT101; Polar; TCS-150	SKT; core temperature; HR; body weight	TSV; TCV; TPV	Linear regression; Physiological Strain Index (PSI)	Thermal sensation vote (TSV)	ANOVA; R-squared; Full recovery time	Northern population (NP) achieve neutral sensation at dMTsk/dt = −0.025; SP at −0.021. NP prone to more discomfort over time.
[48]	China	Both	iButton DS1922L; Polar H10	SKT; HR; HRV	TSV; TCV; TAV	Time-varying exponential models (fitted curves)	Mean SKT, TSV	Prediction accuracy	R^2^: 0.996; 0.997; 0.995 for skin temperature models; >0.85 for TSV models
[49]	China	Indoor	Healink HRV101 Holter	ECG; HRV	TSV; TC	Polynomial regression	Thermal sensation (TS), Thermal comfort (TC)	Prediction accuracy	Strong correlation between mean LF/HF and thermal sensation (R^2^ = 0.9739) and comfort (R^2^ = 1). LF/HF ~1 indicates comfort
[50]	Singapore	Indoor	iButton-DS1992L; Polar H10	SKT; HR	TSV; TP; VP	LGBM; RF; XGB; SVM; KNN; LR; DT	TSV (3-class: cool/neutral/warm); thermal preference (3-class: cooler/no change/warmer); air velocity preference (3-class: less/no change/more air movement)	5-fold cross-validation	Accuracy: 95.6%; AUC: 0.99
[61]	South Korea	Indoor	Empatica E4	EDA; PPG; SKT; HR	TP	LightGBM; XGBoost; Random Forest; CatBoost; Gaussian SVM; Logistic Regression	Thermal preference (3-class: want warmer, comfort, want cooler)	LOSOCV	LightGBM model achieved test accuracy of 79.7% (optimal window size 100 s)
[62]	Spain	Indoor	Samsung Galaxy Watch	HRV	TSV	Random Forest; CNN; LSTM; SVM; MLP; Extra Tree Classifier	TSV (7-point scale) and comfort/discomfort (binary)	LOSOCV and 10-fold CV	Accuracy: 92.2%; MAE = 1.2; MAPE = 20%
[63]	Italy	Indoor	Samsung Galaxy Watch; iButton DS1922	SKT; HRV	TSV	Decision Tree; Multi-layer Perceptron; Adaboost; Random Forest; Extra Tree Regressor; K-Nearest Neighbors; Support Vector Machine	TSV (7-point scale)	LOSOCV	MAE = 0.86, MAPE = 20.9% (Decision Tree on Dataset D2)
[64]	Belgium	Indoor	Polar H7 ECG; gSKIN bodyTEMP patches; gSKIN heat flux patches; Shimmer temperature sensor; Cosinuss One; MetaMAX 3B spiroergometer	HR; SKT; heat flux; aural temperature; metabolic rate	TSV	KNN-LS-SVM; LS-SVM	Thermal sensation (7-point ASHRAE scale reduced to 3-class)	LOSOCV	Accuracy: 87%
[65]	USA	Indoor	Hesvit S3	wrist SKT; wrist skin relative humidity; HR	TSV	ANN (Artificial Neural Network)	TSV (7-point scale)	70/30 train-test split with cross-validation	ANN R^2^ = 0.89; improved overall comfort (over 50% felt neutral after control)
[69]	Italy	Both	iButton Hygrochron	None	TSV	K-means clustering, Spearman’s test	Correlation between daily mean TSV and daily mean normalized temperature	Spearman correlation test with *p* < 0.05 significance level	Only 2 cases showed statistical evidence of correlation between TSV and normalized temperatures
[70]	Ghana	Indoor	iButton Hygrochron	None	TSV; TC; TP	Qualitative triangulation	Simulated indoor temperatures and humidity	Calibration using full-scale experimental buildings	None (proof of concept)
[71]	Japan	Indoor	Thermochron Type-G (KN Laboratories, Inc., Osaka, Japan)	None	25 questions on thermal comfort and adaptive behavior	Field measurement comparison and descriptive statistical analysis	Thermal environment assessment for children; comparison of fixed vs. wearable measurement approaches	Comparison with Japanese Ministry of Health, Labour and Welfare standards	In total, 73% of classroom hours did not meet standards; 50% non-compliance for vertical temperature difference in winter
[72]	Singapore	Indoor	iButton-DS1992L	SKT	TSV; TP; air velocity preference	Statistical analysis (ANOVA, Kruskal–Wallis test)	Thermal comfort, cognitive performance	Prediction accuracy	R^2^: 0.96
[73]	Singapore	Both	Fitbit Ionic smartwatches; iButton Hygrochron DS1923	HR; SKT; skin humidity	TSV; TCV; PAV	Kalman filter; linear regression	Core body temperature; thermal sensation vote (7-point)	LOSOCV	MAE = 0.1 °C (core temp); MAE = 0.3 (TSV); R^2^ = 0.81 (core temp), 0.74 (TSV with humidity)
[74]	China	Indoor	MAXIM iButton DS1922L; Heal Force Prince-100H	SKT; HR	TSV	ACRNN (CNN + GRU + SENet attention); CNN-LSTM; CNN-GRU; LSTM; GRU; CNN; Random Forest; SVM	Thermal sensation (3-class: cold, comfort, hot)	5-fold cross-validation	Overall accuracy: 85.39%; AUC: 0.97 (Cold)—0.96 (Hot)—0.91 (Comfort)
[75]	China	Indoor	iButton DS1922T; Omron HEM-7320; Dimetek DiCare m1C	SKT; HR; BP; HRV	TSV; TCV; thermal satisfaction; thermal expectation; emotion state	Statistical analysis (Pearson correlation, *t*-tests, PMV comparison)	TSV; TCV	Statistical significance testing (*p* < 0.05)	Negative emotion (boring) significantly increased TSV (0.56 scales) and TCV (1.1 scales) during light activities
[76]	China	Indoor	iButton DS1923; Polar H10 Heart Rate Sensor; EMOTIV EPOC X	SKT; HR; HRV; EEG	TSV; TCV; TS; TP; TA	Spearman correlation analysis, matched-sample *t*-tests, linear/polynomial regression fitting	TSV, TCV, Performance Index (PI)	*p*-values for significance, R-squared values for curve fitting	Optimal learning temperature: 24–26 °C. PI showed non-linear correlation with AT (R^2^ = 0.6056). 93% reported comfort at 24 °C target
[77]	Brazil	Indoor	Polar devices (chest and wrist); iButtons DS1923-F5 Hygrochron	HR; SKT	TSV; thermal pleasure; thermal preference	Mixed-effects linear modeling; JOS-3 thermoregulation model simulation	Thermal sensation; thermal pleasure; mean skin temperature; hand temperature	Akaike Information Criterion (AIC); RMSE comparison with JOS-3	JOS-3 captured overall thermal dynamics but underestimated sex-specific distal cooling in females
[78]	Denmark	Indoor	iButton sensors DS1922L-F5#; CCS-103 Careshine Electronic	SKT; HR; HRV	TSV; thermal acceptability; thermal comfort; temperature preference; perceived air quality; visual-analog scales for health symptoms	Statistical analysis (ANOVA, *t*-test, Wilcoxon, Friedman)	Thermal sensation, air quality acceptability, physiological indicators	Statistical significance testing	Lower inhaled air temperature improved perceived air quality but did not change whole-body thermal sensation
[79]	Italy	Indoor	Empatica E4; Interaxon MUSE	PPG; EDA; SKT; ACC; EEG	TC; TS	Random Forest; Naïve Bayes; Support Vector Machine	Thermal comfort/discomfort (binary)	10-fold CV	Accuracy: 76.2%
[80]	Italy	Indoor	Empatica E4; Interaxon MUSE	PPG; EDA; SKT; EEG; HRV	TSV	Random Forest; Gradient Boosting; Gaussian Naïve Bayes; K-Nearest Neighbors; Logistic Regression; Decision Tree	Thermal sensation (binary: hot vs. cold)	70/30 train-test split with 10-fold cross-validation and Bayesian optimization for hyperparameter tuning	Accuracy: 86%; AUC: 0.90
[81]	China	Indoor	ErgoLAB BioSensor Human Factors Recorder; smart wearable ear clip sensors; smart wearable chest strap sensors	HRV; ECG; PPG	TSV; thermal satisfaction	Polynomial regression; K-means clustering	Thermal sensation score (TSV), Cognitive performance index (CP)	Mann–Whitney U test (*p* = 0.03 for LF/HF groups)	Nonlinear correlation identified (R^2^ = 0.9282). LF/HF ratio converged to 1.0 for TSV between −1 and 0.
[82]	Germany	Indoor	Empatica E4	EDA; SKT; PPG; motion-based activity	Thermal stress event-mark button	k-means clustering, linear regression	Temporal clusters (morning/afternoon), correlation between EDA and temperature	Kruskal–Wallis H test, Williams’s Test, Bonferroni correction	Correlation between EDA and temperature was greater for afternoon (r = 0.449) than morning (r = 0.332); significant tonic EDA increase after 28 °C
[83]	Japan	Indoor	PPG sensor (wrist and upper arm); digital automatic blood pressure monitor HEM-7051 (OMRON); ultrasonic Doppler blood flowmeter Bidop ES-100V3 (Hadeco)	PPG	None (no comfort survey; bathing experiment)	Regression analysis (correlation between PBCI and S/D ratio)	PPG Blood Circulation Index (PBCI); correlation with S/D ratio of UDF blood flowmeter	Correlation coefficient comparison with ultrasonic Doppler blood flowmeter (UDF) S/D ratio	R^2^: 0.92
[84]	Italy	Indoor	Interaxon Muse2 headband	EEG	Thermal comfort; thermal sensation; thermal preference	MLP (Multi-Layer Perceptron)	Thermal Label (5-class): very cold, cold, neutral, warm, very warm	80/20 train-test split	Accuracy: 90.1%
[85]	China	Indoor	Custom sensing belt system; Zephyr HxM HR Monitor	ECG; HR; RR; multipoint SKT; rectal temperature; respiratory inductance plethysmograph; acceleration; respiratory sinus arrhythmia	Not relevant	Hardware design and signal processing (QRS detection, peak/valley for RSA)	HR, RR, RSA, energy expenditure, skin/rectal temperature	Bland–Altman analysis against laboratory standard instruments	HR correlation r = 0.99; RR correlation r = 0.98; Temp measurements within acceptable error limits (bias < 0.5 °C)
[86]	China	Indoor	ADInstruments PowerLab & LabChart physiological data collection system; MLT422/A (wrist skin temperature); MLT321 (blood oxygen saturation); MLT118F (skin conductance); LTS2060 (blood pressure); LST2020 (pulse rate); MLA2504 (ECG)	SKT; HR; HRV; EDA; BP; SpO_2_; ECG	TSV	BP neural network (ANN); SVM	Thermal sensation vote (7-point ASHRAE scale); thermal state (3-category)	70% training, 15% validation, 15% testing split	Accuracies of 73.9% (7-category) and 95.7% (3-category) for Multiple Physiological Variable-based ANN
[87]	China	Both	ErgoLAB 3.0 Platform smart sensor; Vive pro eye series	HR; HRV; ECG	Thermal comfort evaluation	VR behavioral experiments and correlational modeling (HRV vs. WBGT)	Thermal comfort assessment	Pearson and Spearman correlation analysis; descriptive statistical analysis	Significant negative correlation (r = −0.894) between comfort level and HRV; positive correlation (r = 0.885) between HRV and WBGT
[88]	South Korea	Indoor	Biopac MP160 and ECG100C	ECG; HRV	TCV	Statistical analysis (ANOVA, Independent *t*-tests), PMV-based cases	Mental stress (LF/HF ratio), thermal comfort sensation (TCV)	Shapiro–Wilk test (normality check), Tukey’s post hoc test	Significant differences in PMV-dependent LF/HF ratio for unskilled group between PMV (0) and PMV (2). Skilled group showed higher mental stress and lower comfort sensation
[89]	China	Indoor	PLUX Explorer ECG signal sensor	ECG; HRV	TSV	Statistical fitting	Temperature correlation/Gender difference fitting	Coefficient of determination	rMSSD R^2^ = 0.85, SDNN R^2^ = 0.64, HR R^2^ = 0.78 (temperature rise); rMSSD R^2^ = 0.78, SDNN R^2^ = 0.84, HR R^2^ = 0.78 (temperature drop)
[68]	China	Indoor	Biosignals Plux ECG and EEG sensors	ECG; EEG; HR; HRV	TSV; TCV; TSVlocal; TCVlocal	Segmented correlation analysis, non-parametric statistical tests (Kruskal–Wallis, Spearman)	TSV (7-point); TCV (5-point)	Statistical significance (*p* < 0.05)	Discomfort dropped from 75% to 0–27% with localized heating; RMSSD/Beta are reliable predictors for resting; Alpha for working
[90]	Italy	Indoor	Samsung Galaxy Watch	HRV; SKT	TSV	Random Forest; Naive Bayes; Decision Trees; Support Vector Machine; K-Nearest Neighbors; Bagging; Adaboost	TSV (thermal sensation vote)	5-fold CV	Intrasubject accuracy: 93% (RF) and 94% (NB); Intersubject average accuracy: 63% (Comfort) and 49% (Discomfort)
[91]	Italy	Indoor	Empatica E4; MUSE Interaxon headband	SKT; HR; HRV; EDA; EEG	TSV; TC	Random Forest; Simple Logistic; Gaussian Naïve Bayes; Logistic Regression; Bagging; Support Vector Machine; Decision Table; J48	Thermal sensation (binary: hot/cold, TS > 0 vs. TS < 0)	10-fold CV	Accuracy: 89.93%; AUC: 0.938
[92]	Germany	Indoor	Empatica E4; RespiBAN Professional by biosignalsplux	SKT; HR; HRV; BVP; ECG	TSV; TC; combined thermal sensation and comfort (cTSC)	Extra Tree (ET); SVM	Thermal discomfort (3-class: no/some/severe cold-induced thermal discomfort)	Stratified 10-fold CV (repeated 20 times)	Accuracy: 83.1%; PMV Comparison
[93]	Italy	Indoor	Samsung Galaxy Watch; Lepton 3.5 IR sensor	HRV; SKT	TSV	K-means clustering; hierarchical clustering; DBSCAN	Thermal comfort clusters (binary: comfort/discomfort)	Silhouette score; Davies-Bouldin index; One-way ANOVA	Silhouette score of 0.52 obtained for winter; K-means outperformed Hierarchical and DBSCAN algorithms
[94]	South Korea	Indoor	Empatica E4 Wristband; HEM-7600T Omron automatic sphygmomanometer; FreeStyle Libre 2 Abbott CGMS	EDA; SKT; HR; HRV; BP; BG; salivary cortisol	TSV; TP; STAI	ANN; GBM; DRF	Thermal preference (binary: preference/no preference)	70/30 train-test split with 5-fold cross-validation for hyperparameter tuning	Proposed model including BG and sCOR reached 73.4% accuracy (10% higher than conventional); sCOR was the most significant feature for both genders
[95]	China	Indoor	Custom wireless skin temperature sensor; wireless skin electrodermal activity sensor; PPG Ear Tip Pulse Sensor	SKT; oral temperature; HR; HRV; EDA	TSV; TC; SFI	Stepwise regression	TSV (9-point thermal sensation scale); TC (thermal comfort 7-point scale)	Within-subject analysis with repeated measures	R^2^: 0.99; Models using rate of change in physiological parameters performed better than using absolute measurements
[96]	Canada; Egypt	Indoor	Emotiv-Epocx; Fitbit Versa 2; Oura ring; Temperature Probes	EEG; HRV; SKT	TSV	Repeated measures ANOVA; PMV calculation	Thermal sensation votes (7-point scale); task performance metrics; EEG-derived focus levels	Repeated measures ANOVA across thermal conditions	Significant differences found in ST and HRV across thermal conditions (*p* < 0.05)
[97]	China	Outdoor	Lifesense Mambo2 fitness tracker; Thermochron iButton DS1921H	HR; SKT	TSV; TP; TCV; thermal acceptability	Logistic Regression (binary and ordinal), independent samples *t*-test	Thermal sensation vote (TSV), Thermal comfort vote (TCV)	Multicollinearity and proportional odds assumption testing, Chi-square statistic	HR and MR decreased over the training period. HR and metabolic rate predict thermal sensation for non-local students but not local students
[98]	Thailand	Indoor	BIOPAC MP160 system	SKT; HR; EDA; AF	Thermal comfort satisfaction	DNN (Deep Neural Network); ANN (Artificial Neural Network)	Thermal comfort satisfaction (5-class: too uncomfortable, uncomfortable, neutral, comfortable, very comfortable)	10-fold cross-validation	DNN achieved 90% average precision, recall, and f-measure; outperformed traditional ANN
[99]	Japan	Indoor	NTC thermistors; GSR sensor; Heart rate sensor	SKT; GSR; HR	TSV	SVM; Neural Network; Random Forest	PMV value (continuous thermal comfort)	5-fold CV; time series split validation	SVM had smallest error (MAE = 3.12; RMSE = 4.35)
[100]	Singapore	Indoor	Not stated	SKT; skin conductance; pulse rate; oxygen saturation; systolic blood pressure; diastolic blood pressure	TSV	CNN (Convolutional Neural Network); SVM-RBF; SVM-polynomial; SVM-linear; SVM-sigmoid	Thermal state (discomfort/comfort binary)	80/20 train-test split	CNN accuracy: 93.33%; SVM-RBF accuracy: 90.6%; both outperformed PMV (76.4%)
[101]	China	Outdoor	ErgoLAB Biosensing; YX102 fingertip pulse oximeter	EDA; HR	TSV; HSV; WSV; TCV; URV; PRS	Linear regression, Polynomial fitting, Wilcoxon signed-rank test	TSV (9-point thermal sensation scale); PRS scores; physiological recovery rates	R-squared, *p*-values, paired-sample *t*-tests	R^2^: 0.9377
[102]	Italy	Indoor	Empatica E4 wristband; Interaxon MUSE headband	IBI; EDA; SKT; EEG	TC; TS; TP	Repeated measures ANOVA, Dwass-Steel-Critchlow–Fligner pairwise comparison	Thermal Label (TL)—integrated index combining thermal comfort, thermal sensation, and thermal preference	Statistical correlation and significance analysis (*p*-value < 0.05)	In total, 55 significant features identified; 8 features significant across all three contrast conditions (C/N, C/W, N/W)
[103]	Italy	Indoor	EmotiBit	EDA; SKT	Thermal comfort; visual comfort; acoustic comfort; air quality; productivity	Deterministic data pipeline and statistical inference (Wilcoxon, Friedman, Kendall’s tau)	Physiological-perceptual divergence (PPD)—decoupling between autonomic activation and subjective comfort	Within-subject paired comparisons using Wilcoxon signed-rank test and Friedman test with Holm correction	Marked physiological divergence (+3.2 °C Tskin, +150% EDA) occurred despite perceptual stability (*p* > 0.10) in most domains
[110]	Italy	Indoor	Interaxon MUSE headband	EEG	TSV	Power Spectral Density (PSD) analysis and Independent Component Analysis (ICA)	Thermal sensation (5-point scale from −2 to 2)	Statistical significance testing using Kruskal–Wallis test and Dwass–Steel–Critchlow–Fligner pairwise comparison	Statistically significant differences found in high-frequency bands (beta, gamma) between cold and warm conditions; warm condition correlated with increased alpha/theta
[111]	China	Indoor	Neuracle 8-lead wireless EEG device	EEG	Thermal comfort; thermal sensation; thermal acceptability	Support Vector Machine (SVM)	Thermal comfort/hot discomfort (binary)	10-fold cross-validation	Accuracy: 74.4%
[112]	South Korea	Indoor	DSI-24 (Wearable Sensing, USA)	EEG	TSV	Statistical analysis (Kruskal–Willis, Mann–Whitney U)	Subjective thermal perception groups based on ASHRAE scale responses	Kruskal–Wallis tests followed by post hoc analysis using Bonferroni correction and Mann–Whitney U tests	EEG indices could distinguish thermal perception groups approximately 6 min after experiment start; F3 channel in left frontal lobe was most sensitive
[113]	Iran	Indoor	NeXus-4 system	HRV; LF/HF ratio; RR; EEG (ALPHA, BETA, THETA)	Noise annoyance; fatigue (VAS)	ANOVA with repeated measures; Tukey’s Test	Cognitive accuracy; Reaction time; Physiological changes (HRV, EEG)	Mauchly’s Test of Sphericity; Effect size (ES)	Gender significantly impacts physiological and performance responses; women are more sensitive to noise/warmth.
[114]	Australia	Indoor	B-Alert X24	EEG	TCV; TSV	Independent Component Analysis (ICA) and Power Spectral Density (PSD) analysis; Wilcoxon signed-rank test for trend identification	Neural signatures (EEG patterns) of short-term thermal adaptation phases and alliesthesia	Cross-referenced with previous steady-state studies and validation across different chunk sizes (1, 2, and 5 min)	Short-term adaptation completed by 15–16 min; identified distinct EEG signatures for warm (n = 21) and cool (n = 30) alliesthesia
[121]	USA	Indoor	GENEActiv accelerometer	Physical activity; metabolic rate	Not relevant	Random Forest; Logistic Regression	Extreme heat events (binary)	80/20 train-test split with 10-fold cross-validation	Accuracy: 99%
[122]	Croatia	Indoor	Garmin Move 3	Steps; activity level; metabolic rate	Thermal sensation; thermal satisfaction	Statistical analysis (ANOVA one-way test of significance)	Total energy expenditure (TEE), occupant sensation/satisfaction	Significance results comparing MET to TEE	MET has significant statistical influence on TEE (Sig 0.000); dissatisfaction occurs when CO_2_ approaches 700 ppm
[124]	China	Both	WSZY-1B temperature logger	None	Thermal comfort; thermal acceptability; thermal tolerability; thermal satisfaction	Statistical analysis (ANOVA, Games–Howell, Welch test)	Temperature distribution, thermal environment acceptance	Statistical significance (*p*-value < 0.05)	Residents experienced wide ranges (−16.6 to 30.6 °C) but unanimously accepted the environment
[125]	Italy	Outdoor	Custom wearable sensor system with PMSA003I air quality sensor; K30 CO_2_ sensor; INMP441 microphone; MLX90640 thermal camera; DHT22 thermohygrometer; Wind Sensor MD0550; NanoLambda NSP32m V1 spectrometer; Fisheye OV5647 camera	None	None	Statistical analysis	Numerical model validation (RMSE vs. measured values)	Comparison between ENVI-met simulation outputs and field measurements (wearable + traditional)	RMSE = 1.43–1.49 °C for air temperature; RMSE = 4.97–5.40% for relative humidity; RMSE = 7.97 °C for mean radiant temperature
[126]	Italy	Both	iButton Hygrochron; ActTrust2 watch	SKT	TSV; TCV	SVM; Random Forest	Thermal comfort vote (3-class: uncomfortable/neutral/comfortable)	Prediction accuracy	Accuracy: 83.02%
[127]	China	Indoor	Custom fEMG collection system; iButton	fEMG; forehead SKT	TSV; TCV	Time–frequency–phase analysis, RMS calculation, Phase locking value (PLV)	Thermal comfort assessment	Mann–Whitney test, Kruskal–Wallis test	fEMG RMS increased 108–164% in cold; masseter muscle showed broad frequency spectrum (200–500 Hz)
[128]	Japan	Indoor	iButton DS1922; Fitbit Versa 4	SKT; HR	TSV; TP; TC	Hybrid model (mathematical + machine learning): Random Forest; Neural Networks; SVM; Decision Trees; KNN	TSV (7-point ASHRAE scale)	5-fold cross-validation	Accuracy = 97.78%; AUC = 1.00; PMV comparison
[129]	USA	Both	iButton DS1923; Polar H7; POSH Mobile cell phone	SKT; HR; accelerometry	TSV; TP	Stochastic Gradient Boosting (gbm); Random Forest by Randomization (extraTrees); C5.0; Random Forest (rf); Neural Network (nnet); SVM; Logistic Regression; Linear Discriminant Analysis; K-Nearest Neighbors; Naive Bayes; Classification and Regression Trees (rpart); J48 Decision Tree; Rule-Based Classifier (PART); Bagged Classification and Regression Trees (treebag)	Thermal preference (3-class: Cooler, No change, Warmer)	5-fold CV repeated 20 times	Accuracy: 78%; AUC: 0.79; PMV Comparison
[130]	China	Indoor	MAXIM iButton DS1922L; Prince-100H oximeter	SKT; HR	STC	AGRU (Attention-based Gated Recurrent Unit); GRU; LSTM; CNN; RNN; RF; SVM; DT	STC (sleep thermal comfort)—3-class: cold-discomfort, comfort, hot-discomfort	5-fold CV	Average accuracy of 86.99%, macro-F1 score of 0.87
[131]	USA	Indoor	Empatica E4; UbiBot WS1	SKT; HR; EDA; ACC	TP; TSV	Ranger (Random Forest); XGBoost; SVM; KNN; decision trees; cforest; lda; glm; nb	Thermal preference (3-class: want warmer; no change; want cooler)	5-fold CV	Accuracy: 98.95%
[132]	Australia	Indoor	Empatica E4	EDA; BVP; HR; HRV; SKT; 3-axis acceleration	TSV; TP; clothing level	Logistic Regression (simple and multiple)	Air-conditioner switching probability (heating/cooling)	None	Not validated (provided as illustrative usage)
[133]	South Korea	Indoor	Empatica E4	BVP; EDA; SKT; HR	TSV	CNN-SVM hybrid (ensemble transfer learning); CNN-RF hybrid; DNN-SVM hybrid; DNN-RF hybrid	TSV (3-class: cool, neutral, warm)	80/20 train-test split	Accuracy: 95%; F1-score = 95%
[134]	USA	Indoor	Empatica E4	HR; BVP; SKT; EDA	TSV; thermal satisfaction; thermal acceptability	ANN; SVM	Thermal sensation (7-point scale); thermal satisfaction (7-point scale); thermal acceptance (binary)	10-fold CV	ANN Acceptance F1-score: 0.669; SVM better for physiological data
[135]	USA	Indoor	Garmin Vivosmart 3; Empatica Embrace 2	HR; HRV; EDA; SKT; stress level	IAQ evaluation; thermal comfort evaluation	Random Forest	IAQ evaluation (7-point scale); thermal comfort evaluation (7-point scale)	5-fold cross-validation with time-series split; 20% hold-out test set; leave-one-subject-out cross-validation	84.76% accuracy for IAQ (RMSE = 0.284; MAE = 0.216; R^2^: 0.847), 70.5% for thermal comfort. Outperformed general models (69.7% and 64.3%)
[136]	USA	Indoor	Empatica E4	SKT; HR; skin conductance; 3-axis accelerometer	TSV; TP	Random Forest; Gradient Boosting; k-NN; SVM; Learning Vector Quantization	TSV (7-point ASHRAE scale)	10-fold CV	Accuracy: 91.76%; Inclusion of activity-based metabolic rate improved performance by up to 8.5%
[67]	Japan	Indoor	Empatica E4	SKT; HR; EDA	TSV	Random Forest; Logistic Regression; Support Vector Machine; k-Nearest Neighbor	TSV (7-point)	LOSOCV	Accuracy F1 = 0.85
[137]	Serbia	Indoor	E66 Smart Bracelet	SKT; HR	TSV	Statistical correlation analysis	Thermal sensation vote	Correlation analysis for individual users	0.54 female, 0.36 male correlation between skin temperature and comfort
[138]	Philippines	Indoor	LM35 Temperature Sensor; Huawei GT2e smartwatch	SKT; HR; stress level	TSV	CBE thermal comfort tool (PMV/PPD) and rule-based logic	PMV, PPD, A/C Thermostat setpoint	PMV Comparison	Accuracy: 96.67% (subjective vs. objective match)
[139]	USA	Indoor	Fitbit Charge HR; Microsoft Smart Band 2	HR; metabolic rate; SKT; skin resistance	TCV	ANN Model, Non-linear fitting	Metabolic rate (MET), comfort vote	Prediction accuracy	Metabolic rate varies significantly throughout the day, impacting PMV accuracy by over 2.5 points
[140]	South Korea	Indoor	Fitbit Charge 2	HR	Not relevant	Deep Learning CNN (HRNet); AlexNet-based convolutional neural network	Metabolic rate (MET)	70/30 train-test split; individual-specific training (self-prediction) vs. third-party prediction	Accuracy rate 77–89% (self-prediction), 65% (third-party prediction)
[141]	Japan	Indoor	Fitbit Versa 4; iButton DS1922L	SKT; HR	TSV; TP	SMAT (Selective Multi-source Adaptation for Thermal comfort); DSAN; CNN-LSTM	Thermal preference (3-class: want cooler, no change, want warmer)	LOSOCV	Average accuracy of 71.00% in field and 88.71% in climate chamber for SMAT model
[142]	South Korea	Both	Apple Watch	HR; HRV; blood oxygen saturation; wrist SKT	TP	Cross-scale data convergence	Occupant behavioral/comfort patterns at the urban scale	Preliminary correlation analysis (Pearson correlation matrix)	Outdoor temperature correlated best with daily step count/walking distance; 62% preference for “no change” in mild conditions
[143]	Singapore	Both	Apple Watch Series 3 or newer	HR; resting HR; blood oxygen saturation; step count; walking distance; stand time	Noise distraction; thermal preference	Ecological Momentary Assessment (EMA)	User satisfaction/distraction feedback	Longitudinal field study data analysis	2400 surveys collected; noise distraction experienced 58% of the time
[144]	Singapore	Both	Apple Watch	HR; oxygen saturation; stepcount; walking distance; stand time	TP; noise distraction; noise source identification	Statistical analysis	Noise distraction (3-class: None/A little/A lot); noise source (7-class: talking/traffic/weather/appliances/construction/other); thermal preference (3-class: no change/warmer/cooler)	Correlation analysis	Significant correlations (*p* < 0.05)
[145]	Singapore	Outdoor	Apple Watch	HR; respiratory rate; blood pressure; blood oxygen saturation; activity data; walking speed; step count	TSV	Urban digital twin (UDT), SVI semantic segmentation (Mask2Former)	Real-time thermal sensation, solar position mapping	Proof of concept/visualization	Identified trends where open space areas correlate with high temperature and hot perception
[146]	Singapore	Both	Apple Watch; Kestrel Drop D2; Tile GPS tracker	HR; step count	TSV; TP; thermal sensation; heat stress	Cramer’s V correlation, UTCI calculation	UTCI stress category	Correlation comparison between UTCI and subjective sensation	Cramer’s V = 0.262 (weak association between UTCI and older adults’ perception)
[147]	Singapore	Both	Apple Watch	HR; blood oxygen; step count	Thermal preference; noise distraction	None (proof of concept)	None (proof of concept)	None (proof of concept)	None (proof of concept)
[148]	Iran	Indoor	Apple Watch Series 9	HR; SKT	Thermal sensation scale	Two-way repeated measures ANOVA, Pearson’s correlation, Paired *t*-tests	Thermal comfort boundaries for sleep	Statistical significance testing (95% confidence level)	Strong correlation between bed surface temp and heart rate (*p* = 0.001); gender-specific comfort thresholds established
[149]	China	Indoor	Thermocouples; Powerlab 8/30 system with ML-1340 leads	SKT; HRV	TSV; TC	Linear/polynomial regression, HRV spectral analysis	Thermal sensation vote, comfort vote	Paired *t*-tests, ANOVA	Female comfortable operative temperature (26.3 °C) higher than male (25.3 °C); females more sensitive to temp; skin temp is good predictor below neutrality
[150]	Thailand	Indoor	BIOPAC Systems MP36R	SKT; HR; EDA; airflow	Thermal comfort satisfaction	Decision Tree; k-nearest neighbors; Artificial Neural Network; Support Vector Machine; Naive Bayes	Thermal satisfaction (5-class: extremely uncomfortable; uncomfortable; neutral; comfortable; extremely comfortable)	10-fold CV	Accuracy: F1 = 90%
[151]	Japan	Indoor	NTC Thermistors; Pulse Sensor	SKT; HR	PMV questionnaire asking temperature change preference	Random Forest; Neural Network; SVM	Subjective thermal comfort (7-point scale); subjective thermal comfort (3-point scale); objective thermal comfort (PMV)	5-fold CV	MAE: 0.73 (RF, 7-level subjective), MAE: 0.47 (NN, objective), F1-score: 0.79 (RF, 3-level subjective)
[152]	South Korea	Indoor	Thermocouple T-type	SKT	Not relevant	J48 decision tree; stepwise regression	Clothing thermal insulation (clo value)	70/30 train-test split	Accuracy: 88.04%; R^2^: 0.7380
[153]	USA	Indoor	Custom wristband with MLX90614 contact-less infrared temperature sensor	SKT	TSV; thermal satisfaction	KNN; Random Forest; SVM; Decision Trees	TSV (3-class: cold, comfortable, hot); thermal satisfaction (binary: satisfied, dissatisfied)	5-fold CV	Environmental sensors achieved highest accuracy (90% regular, 84% with PCS)
[154]	USA	Indoor	Custom wristband with MLX90614 contact-less infrared temperature sensor	SKT	TSV; thermal satisfaction	SVM (quadratic kernel); Random Forest; KNN; Subspace KNN; Subspace Discriminant	Thermal sensation (3-class: cold, comfortable, hot); thermal satisfaction (2-class: satisfied, dissatisfied); combined thermal sensation and satisfaction (6-class)	Within-subject 5-fold CV	Ambient air temp accuracy: 81%; wrist temp accuracy: 76%; camera accuracy: 75%; combined accuracy: 85%
[155]	Mexico	Indoor	Custom IoT smartwatch (XIAO ESP32C3 microcontroller; GY-906 temperature sensor; MAX30102 heart rate sensor; XIAO Round Display)	SKT; HR	TSV; thermal acceptance; clothing insulation level; metabolic activity; location	Open ecosystem for data collection	Thermal sensation, thermal acceptance	Calibration against reference instruments	MAE skin temperature 0.41 °C, MAE HR 3.6 bpm
[156]	USA	Indoor	Custom wristband devices (BASIS)	SKT; HR; perspiration rate	PMV	Artificial Neural Networks (ANN)	PMV index	75% training, 20% validation, 5% testing split	RMSE = 0.023; R^2^: 0.999 with Ground Truth PMV
[157]	USA	Indoor	Custom wristband devices (BASIS)	SKT; HR; skin conductivity	PMV	Artificial Neural Networks (ANN)	Predicted PMV value	Correlation analysis	R^2^: 99.9% to traditional PMV estimates
[158]	South Korea	Indoor	Custom wrist band with NTC thermistors	SKT	TSV	Multiple Linear Regression (Stepwise)	TSV (9-point thermal sensation scale from −4 to +4)	Train-test split (422 datasets for training, 418 for validation)	RMSE = 0.95 ± 0.21 (personal model with fingertip and 3 wrist regions); R^2^: 0.72 (general model), 0.92 (personal model)
[159]	South Korea	Indoor	Custom smart face mask with MLX90614-DCC (infrared temperature sensor); ear clip plethysmography sensor; NXFT15XH103FA2B130 (thermistor); DHT-22	SKT; HR; EBT	TSV	Random Forest	TSV (7-point scale)	70/30 train-test split	Overall accuracy 77.45% without heart rate feature; EBT significantly correlates with TSV
[160]	Greece	Indoor	Custom wrist-worn device with STM32WL55 SoC; Maxim MAX86141; Kionix KX-122; Maxim MAX32664	HR; SpO_2_; HRV; respiratory rate	PMV	Simulation (CFD), regression (MRT), machine learning (Random Forest for clothing prediction)	Thermal comfort (PMV index)	Validation of PMV results via correlation with HRV biological indicators	Shift in TC precipitates decrease in HRV indices (SDNN avg decrease ~6.18%, RMSSD ~6.67% for discomfort)
[161]	Italy	Indoor	Custom low-cost PPG sensor; commercial smart strap with ECG electrodes	HR	PMV	linear/polynomial regression (Relationship between HR and Metabolic Rate)	Metabolic rate (met); PMV index	validation against reference piezoelectric sensor system	R^2^: 0.985
[162]	Greece	Indoor	Custom wearable device with ESP-WROOM-32 module	HR	PMV	Predicted Mean Vote (PMV) algorithm with manual profile enhancement	PMV (7-point thermal comfort scale)	Comparison of predicted PMV to stated perceived thermal sensation	Results were ‘satisfactory’ over 20 h of data, though population sample needs expansion
[163]	Japan	Indoor	SHT15, Sensirion AG; SHTDL-3 System, SysCom Corp.	SKT; core temperature	TSV; TP; TC; perspiration sensation	Mass transfer theory (Sherwood/Reynolds numbers), Human thermal load method	Sweat evaporation rate; evaporative heat loss	Comparison with weight-loss measurements; Bland–Altman analysis	Predicted trend matched weight-loss data with MAE = 5.7 g; 94% agreement in Bland–Altman
[164]	South Korea	Indoor	Custom watch-type sweat rate sensor	sweat rate	Thermal sensation vote	Linear fitting, heat circuit modeling	Sweat rate (g/m^2^h), thermal status stage	Comparison with artificial skin (wet-cup method)	Sensitivity of 0.039 (pF/s)/(g/m^2^h), linearity 97.9%, error less than 10% under 1.5 m/s air velocity
[165]	South Korea	Outdoor	myBeat Union Tool; Terumo P2100	HR; HRV; respiratory rate; blood pressure; pulse rate	PMV; PPD; TSV; CSV; POMS; SD	Statistical analysis (Independent *t*-tests, paired *t*-tests, Wilcoxon signed-rank)	Thermal comfort, physiological/psychological relaxation	Correlation strength	*p* < 0.05
[166]	China	Indoor	JAALEE JHT-2/JHT-2P	SKT; skin humidity	Thermal comfort scale	RF-IBWO-BPNN-LSTM; LSTM; BWO-LSTM; RF-LSTM; ANN; SVM	Thermal comfort (9-point scale: −4 to +4, categorized into cold/neutral/hot)	70/30 train-test split	Average accuracy exceeded 98%; F1-score: 0.95
[167]	USA	Indoor	Microsoft Band 1; Microsoft Band II	HR; SKT; calories consumed; metabolic rate	TSV	Ridge regression with transfer active learning	TSV (7-point scale: −3 to +3)	50/50 train-test split per day per user; 5-fold cross-validation for hyperparameter tuning	RMSE = 0.818 ± 0.05
[66]	Singapore	Indoor	Microsoft Band 2	HR; SKT	TSV	Intelligent Thermal Comfort Neural Network (ITCNN)	Thermal comfort score	10-fold cross-validation	MAE = 0.81–0.85; outperforms PMV model (MAE = 0.97) by 13.1% on average
[171]	China	Outdoor	YX102 finger clip heart rate meter; TB-300 infrared tympanic thermometer; wireless universal anemometer and temperature recorder	HR; ACT; FT; SpO_2_	TSV; Tpref; TP	Linear regression, Probit regression, Physiological Stress Warning Index (PWI)	MTSV, Preferred PET/UTCI, PWI (Physiological Stress Warning Index)	Spearman rank correlation, R-squared, gray correlation method	Neutral PET in shade: 21.4 °C; neutral UTCI: 27.2 °C; exercise in sunlight should be avoided if PET > 37.8 °C or UTCI > 38.2 °C
[172]	South Korea	Outdoor	Technox LT-8 A; UNION TOOL WHS-1; Terumo P2100	Forehead SKT; HR; HRV; blood pressure; pulse rate	TSV; CSV	Fanger’s PMV model, Statistical analysis (Welch’s *t*-test, Wilcoxon signed-rank test)	Predicted Mean Vote (PMV), Predicted Percentage of Dissatisfied (PPD)	Comparison with subjective evaluation (TSV/CSV)	Discrepancy found in spring where MRT excessively influenced PMV/PPD compared to subjective votes
[177]	USA, Singapore, Germany	Indoor	Microsoft Health Band	GSR; SKT	Thermal comfort	OccuTherm	Thermal comfort level	None	Dataset provides information to test the hypothesis that tall/skinny people tolerate heat better
[184]	China	Indoor	WitMotion BWT61CL	IMU 9-axis: 3-axis acceleration; 3-axis angular velocity; 3-axis Euler angles	None (activity recognition only)	Fully Connected Neural Network (FCNN), 1D-CNN	Thermal comfort-related activity category (30 classes: 10 cold-related, 10 heat-related, 10 neutral)	LOSOCV	Accuracy: 93.07% offline (1D-CNN); ~90% online
[185]	China	Indoor	HES-S3; HXM-08L	SKT; HR	TSV; TCV; TPV	Multiple linear regression; online recursive correction method	Thermal sensation vote (TSV)	Cross-validation (80/20)	RMSE decreased from 1.182 to 0.652 after five real-time corrections; accuracy improved by 44.8%
[186]	Japan	Indoor	Silmee Pro W-22	energy expenditure; SKT; HR; pulse; pulse wave peak interval (PPI)	TSV	Regression and correlation analysis; CBE thermal comfort tool (PMV)	Predicted Mean Vote (PMV), thermal sensation vote (TSV)	Pearson correlation and linear regression (comparing TSV to standard vs. actual PMV)	R^2^: 0.2218; standard PMV correlated better (rho = 0.44) with TSV than PMV
[187]	Malaysia	Indoor	Finger heart rate monitor; MAX30102 heart rate sensor	HR; SpO_2_	PMV	Regression analysis	PMV (thermal comfort scale −3 to +3)	*p*-value significance test	Heart rate *p*-value = 0.03262 (statistically significant); humidity *p*-value = 0.676 (less significant)
[188]	China	Indoor	KF1 sensor; 16-channel cognitive biological feedback instrument; RT-72ui data logger	SKT; HR; HRV; BVP	TSV; TCV; thermal acceptability; thermal preference	Statistical analysis (independent sample *t*-test, paired-sample *t*-test)	TSV, TCV	Comparison between N and S groups	S group participants showed better heat tolerance and wider adaptable zones; mean skin temp failed to predict sensation in step changes
